# Multicriteria Decision-Making Approach with Hesitant Interval-Valued Intuitionistic Fuzzy Sets

**DOI:** 10.1155/2014/868515

**Published:** 2014-03-27

**Authors:** Juan-juan Peng, Jian-qiang Wang, Jing Wang, Xiao-hong Chen

**Affiliations:** ^1^School of Business, Central South University, Changsha 410083, China; ^2^School of Economics and Management, Hubei University of Automotive Technology, Shiyan 442002, China

## Abstract

The definition of hesitant interval-valued intuitionistic fuzzy sets (HIVIFSs) is developed based on interval-valued intuitionistic fuzzy sets (IVIFSs) and hesitant fuzzy sets (HFSs). Then, some operations on HIVIFSs are introduced in detail, and their properties are further discussed. In addition, some hesitant interval-valued intuitionistic fuzzy number aggregation operators based on *t*-conorms and *t*-norms are proposed, which can be used to aggregate decision-makers' information in multicriteria decision-making (MCDM) problems. Some valuable proposals of these operators are studied. In particular, based on algebraic and Einstein *t*-conorms and *t*-norms, some hesitant interval-valued intuitionistic fuzzy algebraic aggregation operators and Einstein aggregation operators can be obtained, respectively. Furthermore, an approach of MCDM problems based on the proposed aggregation operators is given using hesitant interval-valued intuitionistic fuzzy information. Finally, an illustrative example is provided to demonstrate the applicability and effectiveness of the developed approach, and the study is supported by a sensitivity analysis and a comparison analysis.

## 1. Introduction

Since fuzzy sets were proposed by Zadeh [1], the studies on multicriteria decision-making (MCDM) problems have made great progress. Further, fuzzy sets were generalized to intuitionistic fuzzy sets (IFSs) by Atanassov [2, 3], where each element in an IFS has a membership degree and a nonmembership degree between 0 and 1, respectively. Then, Atanassov and Gargov [4] proposed the notion of interval-valued intuitionistic fuzzy sets (IVIFSs) which are the extension of IFSs, where the membership degree and nonmembership degree of an element in an IVIFS are, respectively, represented by intervals in [0,1] rather than crisp values between 0 and 1. In recent years, many researchers have studied the theory of IVIFSs and applied it to various fields [5–8]. For instance, Atanassov [9] introduced the operators of IVIFSs. Lee [10] proposed a method for ranking interval-valued intuitionistic fuzzy numbers (IVIFNs) for fuzzy decision-making problems. Lee [11] provided an enhanced MCDM method of machine design schemes under the interval-valued intuitionistic fuzzy environment. Li [12] proposed a TOPSIS based nonlinear-programming method for MCDM problems with IVIFSs. Park et al. [13] extended the TOPSIS method to solve group MCDM problems in interval-valued intuitionistic fuzzy environment in which all the preference information provided by decision-makers is presented as IVIFNs. Chen et al. [14] developed an approach to tackle group MCDM problems in the context of IVIFSs. Nayagam and Sivaraman [15] introduced a method for ranking IVIFSs and compared it to other methods by means of numerical examples. Chen et al. [16] presented a MCDM method based on the proposed interval-valued intuitionistic fuzzy weighted average (IVIFWA) operator. Meng et al. [17] developed an induced generalized interval-valued intuitionistic fuzzy hybrid Shapley averaging (GIVIFHSA) operator and applied it to MCDM problems.

Hesitant fuzzy sets (HFSs), another extension of traditional fuzzy sets, provide a useful reference for our study under hesitant fuzzy environment. HFSs were first introduced by Torra and Narukawa [18], and they permit the membership degrees of an element to be a set of several possible values between 0 and 1. HFSs are highly useful in handling the situations where people have hesitancy in providing their preferences over objects in the decision-making process. Some aggregation operators of HFSs were studied and applied to decision-making problems [19–21]. Then, the correlation coefficients of HFSs, the distance measures, and correlation measures of HFSs were discussed [22–24], based on which Peng et al. [25] presented a generalized hesitant fuzzy synergetic weighted distance measure. Zhang and Wei [26] developed the E-VIKOR method and TOPSIS method to solve MCDM problems with hesitant fuzzy information. Zhang [27] developed a wide range of hesitant fuzzy power aggregation operators for hesitant fuzzy information. Chen et al. [28] generalized the concept of HFSs to hesitant interval-valued fuzzy sets (HIVFSs) in which the membership degrees of an element to a given set are not exactly defined but denoted by several possible interval values. Wei [29] defined HIVFSs and some hesitant interval-valued fuzzy aggregation operators. Wei and Zhao [30] developed some Einstein operations on HIVFSs and the induced hesitant interval-valued fuzzy Einstein aggregation (HIVFEA) operators and applied them to MCDM problems. Zhu et al. [31] defined dual HFSs (DHFSs) in terms of two functions that return two sets of membership degrees and nonmembership degrees rather than crisp numbers in HFSs. If the idea of dual HFSs is used from a new perspective, then another extension of HFSs may be defined in terms of one function that the element of HFSs returns a set of IFSs, which are called hesitant intuitionistic fuzzy sets (HIFSs). But decision-makers usually cannot estimate criteria values of alternatives with exact numerical values when the information is not known precisely. Therefore, interval values in fuzzy sets can represent it better than specific numbers, such as interval-valued fuzzy sets (IVFSs) and IVIFSs. Furthermore, although the theories of IVIFSs and HFSs have been developed and generalized, they cannot deal with all sorts of uncertainties in different real problems. For example, when we ask the opinion of an expert about a certain statement, he or she may answer that the possibility that the statement is true is [0.1, 0.2] and that the statement is false is [0.4, 0.5], or the possibility that the statement is true is [0.5, 0.6] and that the statement is false is [0.3, 0.5]. This issue is beyond the scope of IVFSs and IVIFSs. Therefore, some new theories are required.

So the concept of hesitant interval-valued intuitionistic fuzzy sets (HIVIFSs) is developed in this paper. Comparing to the existing fuzzy sets mentioned above, HIVIFSs are a new extension of HFSs, which support a more flexible and simpler approach when decision-makers provide their decision information in a hesitant interval-valued intuitionistic fuzzy environment. Furthermore, IVIFSs, HFSs, HIVFSs, and HIFSs are all the special cases of HIVIFSs.

In this paper, HFSs are extended based on IVIFSs. HIVIFSs are defined, and their properties and applications are also discussed. Thus, the rest of this paper is organized as follows. In Section 2, the definitions and properties of IVIFSs and HFSs are briefly reviewed. In Section 3, the notion of HIVIFSs is proposed, and the operations and properties of HIVIFSs based on *t*-conorms and *t*-norms are discussed. In Section 4, some hesitant interval-valued intuitionistic fuzzy number aggregation operators are developed and applied to MCDM problems.  Section 5 gives an example to illustrate the application of the developed method. Finally, the conclusions are drawn in Section 6.

## 2. Preliminaries

In this section, some basic concepts and definitions related to HIVIFSs are introduced, including interval numbers, IVIFSs, and HFSs. These will be utilized in the subsequent analysis.

### 2.1. Interval Numbers and Their Operations

**Definition 1 (see [32–34])**

Let $\tilde{a}=[a^{L},a^{U}]=\{x|a^{L}\leq x\leq a^{U}\}$; then $\tilde{a}$ is called an interval number. In particular, if 0 ≤ *a* ^*L*^ ≤ *x* ≤ *a* ^*U*^, then $\tilde{a}$ is reduced to a positive interval number.

Consider any two interval fuzzy numbers $\tilde{a}=[a^{L},a^{U}]$ and $\tilde{b}=[b^{L},b^{U}]$, and their operations are defined as follows:

1. $\tilde{a}=\tilde{b}\Leftrightarrow a^{L}=b^{L},a^{U}=b^{U}$;
2. $\tilde{a}+\tilde{b}=\left[a^{L}+b^{L},a^{U}+b^{U}\right]$;
3. $\tilde{a}-\tilde{b}=[a^{L}-b^{U},a^{U}-b^{L}]$;
4. $\tilde{a}\times \tilde{b}=[\min\left\{a^{L}b^{L},a^{L}b^{U},a^{U}b^{L},a^{U}b^{U}\right\}$, max⁡⁡{*a* ^*L*^ *b* ^*L*^, *a* ^*L*^ *b* ^*U*^, *a* ^*U*^ *b* ^*L*^, *a* ^*U*^ *b* ^*U*^}];
5. $k\tilde{a}=[ka^{L},ka^{U}],\;k>0$.

### 2.2. IVIFSs

Atanassov first proposed IFSs, being enlargement and development of Zadeh's fuzzy sets. IFSs contain the degree of nonmembership, which makes it possible for us to model unknown information. The definition of IVIFSs given by Atanassov and Gargov [4] is shown as follows.

**Definition 2 (see [4])**

Let *D*[0,1] be the set of all closed subintervals of the interval [0,1]. Let *X* be a given set and *X* ≠ *⌀*. An IVIFS in *X* is an expression given by *A* = {〈*x*, *μ*_*A*_(*x*), *ν*_*A*_(*x*)〉 | *x* ∈ *X*}, where *μ*_*A*_ : *X* → *D*[0,1], *ν*_*A*_ → *D*[0,1] with the condition 0 < sup_*x*_ *μ*_*A*_(*x*) + sup_*x*_ *ν*_*A*_(*x*) ≤ 1. The intervals *μ*_*A*_(*x*) and *ν*_*A*_(*x*) denote the degree of belongingness and nonbelongingness of the element *x* to the set *A*, respectively. Thus, for each *x* ∈ *X*, *μ*_*A*_(*x*) and *ν*_*A*_(*x*) are closed intervals whose lower and upper boundaries are denoted by *μ*_*A*_^*L*^(*x*), *μ*_*A*_^*U*^(*x*) and *ν*_*A*_^*L*^(*x*), *ν*_*A*_^*U*^(*x*), respectively, and then

(1) $$\begin{array}{c} A=\left\{\langle x,\left[\mu_{A}^{L}\left(x\right),\mu_{A}^{U}\left(x\right)\right],\left[\nu_{A}^{L}\left(x\right),\nu_{A}^{U}\left(x\right)\right]\rangle \;|\;x\in X\right\}, \end{array}$$

where 0 < *μ*_*A*_^*U*^(*x*) + *ν*_*A*_^*U*^(*x*) ≤ 1, *μ*_*A*_^*L*^(*x*) ≥ 0, *ν*_*A*_^*L*^(*x*) ≥ 0. For each element *x*, the hesitancy degree can be calculated as follows: Π_*A*_(*x*) = 1 − *μ*_*A*_(*x*) − *ν*_*A*_(*x*) = [1 − *μ*_*A*_^*U*^(*x*) − *ν*_*A*_^*U*^(*x*), 1 − *μ*_*A*_^*L*^(*x*) − *ν*_*A*_^*L*^(*x*)]. The set of all IVIFSs in *X* is denoted by IVIFS(*X*). An interval-valued intuitionistic fuzzy number (IVIFN) is denoted by *A* = ([*a*, *b*], [*c*, *d*]) and the degree of hesitance is denoted by [*e*, *f*] = [1 − *a* − *d*, 1 − *a* − *c*] for convenience.

**Definition 3 (see [16])**

Let ${\tilde{\alpha }}_{i}=\langle \left[a_{i},b_{i}],[c_{i},d_{i}\right]\rangle \;(1\leq i\leq n)$ be a collection of IVIFNs and let *w*_*i*_  (1 ≤ *i* ≤ *n*) be the crisp values, where ${\tilde{\alpha }}_{i}=\langle \left[a_{i},b_{i}],[c_{i},d_{i}\right]\rangle =[[a_{i},b_{i}],[1-d_{i},1-c_{i}]]$, 0 ≤ *a*_*i*_ ≤ *b*_*i*_ ≤ 1, 0 ≤ *c*_*i*_ ≤ *d*_*i*_ ≤ 1, 0 ≤ *b*_*i*_ + *d*_*i*_ ≤ 1, and 1 ≤ *i* ≤ *n*, and then the interval-valued intuitionistic fuzzy weighted average operator can be defined as follows:

(2) IVIFWAw(α~1,α~2,…,α~n) =∑i=1n[[ai,bi],[1−di,1−ci]]×wi∑i=1nwi =[[∑i=1naiwi∑i=1nwi,∑i=1nbiwi∑i=1nwi],[∑i=1n(1−di)wi∑i=1nwi,∑i=1n(1−ci)wi∑i=1nwi]] =[[a~,b~],[c~,d~]],

where $\text{IVIFW}{\text{A}}_{w}({\tilde{\alpha }}_{1},{\tilde{\alpha }}_{2},\ldots ,{\tilde{\alpha }}_{n})=[[\tilde{a},\tilde{b}],[\tilde{c},\tilde{d}]]=\langle \left[\tilde{a},\tilde{b}],[1-\tilde{d},1-\tilde{c}\right]\rangle$ is an interval-valued intuitionistic fuzzy value;$\tilde{a}$, $\tilde{b}$, $\tilde{c}$, and $\tilde{d}$ are calculated by the Karnik-Mendel algorithms [35].

**Example 4**

Let ${\tilde{\alpha }}_{1}=\langle [0.3,0.6],\left[0.1,0.2\right]\rangle$ and ${\tilde{\alpha }}_{2}=\langle \left[0.4,0.6\right],\left[0.1,0.3\right]\rangle$ be two IVIFNs, and *w*_1_ = 0.3, *w*_2_ = 0.5. According to (2),

(3) IVIFWAw(α~1,α~2) =[[0.3×0.3+0.4×0.50.3+0.5,0.6×0.3+0.6×0.60.3+0.5],     [(1−0.2)×0.3+(1−0.3)×0.50.3+0.5,  (1−0.1)×0.3+(1−0.1)×0.50.3+0.5]] =[[0.3625,0.6750],[0.7375,0.9000]] =〈[0.3625,0.6750],[1−0.9000,1−0.7375]〉 =〈[0.3625,0.6750],[0.1000,0.2625]〉.

**Definition 5 (see [36])**

Let $\tilde{\alpha }=\langle \left[a,b],[c,d\right]\rangle$ be an IVIFN, and then an accuracy function $L(\tilde{\alpha })$ can be defined as follows:

(4) $$\begin{array}{c} L\left(\tilde{\alpha }\right)=\frac{a+b-d\left(1-b\right)-c\left(1-a\right)}{2}, \end{array}$$

where $L(\tilde{\alpha })\in [-1,1]$ and 1 ≤ *i* ≤ *n*.

**Definition 6 (see [36])**

Let ${\tilde{\alpha }}_{1}$ and ${\tilde{\alpha }}_{2}$ be two IVIFNs, and then the following comparison method must exist.

1. If $L({\tilde{\alpha }}_{1})>L({\tilde{\alpha }}_{2})$, then ${\tilde{\alpha }}_{1}>{\tilde{\alpha }}_{2}$.
2. If $L({\tilde{\alpha }}_{1})=L({\tilde{\alpha }}_{2})$, then ${\tilde{\alpha }}_{1}={\tilde{\alpha }}_{2}$.

**Example 7**

Let ${\tilde{\alpha }}_{1}=\langle \left[0.4,0.6\right],\left[0.1,0.2\right]\rangle$ and ${\tilde{\alpha }}_{2}=\langle \left[0.5,0.6\right],[0.2,0.3]\rangle$ be two IVIFNs. According to (4), $L({\tilde{\alpha }}_{1})=\left(0.4+0.6-0.2\times (1-0.6)-0.1\times (1-0.4)\right)/2=0.43$ and $L({\tilde{\alpha }}_{2})=0.44$. $L({\tilde{\alpha }}_{2})>L({\tilde{\alpha }}_{1})$ can be obtained, so the optimal one(s) is ${\tilde{\alpha }}_{2}$.

**Definition 8 (see [37–39])**

A function *T* : [0,1]×[0,1]→[0,1] is called *t*-norm if it satisfies the following conditions:

1. for all *x* ∈ [0,1], *T*(1, *x*) = *x*;
2. for all *x*, *y* ∈ [0,1], *T*(*x*, *y*) = *T*(*y*, *x*);
3. for all *x*, *y*, *z* ∈ [0,1], *T*(*x*, *T*(*y*, *z*)) = *T*(*T*(*x*, *y*), *z*);
4. if *x* ≤ *x*′, *y* ≤ *y*′, then *T*(*x*, *y*) ≤ *T*(*x*′, *y*′).

**Definition 9 (see [37–39])**

A function *S* : [0,1]×[0,1]→[0,1] is called *t*-conorm if it satisfies the following conditions:

1. for all *x* ∈ [0,1], *S*(0, *x*) = *x*;
2. for all *x*, *y* ∈ [0,1], *S*(*x*, *y*) = *S*(*y*, *x*);
3. for all *x*, *y*, *z* ∈ [0,1], *S*(*x*, *S*(*y*, *z*)) = *S*(*S*(*x*, *y*), *z*);
4. if *x* ≤ *x*′, *y* ≤ *y*′, then *S*(*x*, *y*) ≤ *S*(*x*′, *y*′).

There are some well-known Archimedean *t*-conorms and *t*-norms [39, 40].

(1) Let *k*(*t*) = −In⁡*t*, *l*(*t*) = −In⁡(1 − *t*), *k* ^−1^(*t*) = *e* ^−*t*^, *l* ^−1^(*t*) = 1 − *e* ^−*t*^, and then algebraic *t*-conorms and *t*-norms are obtained as follows: *T*(*x*, *y*) = *xy*, *S*(*x*, *y*) = 1 − (1 − *x*)(1 − *y*).
(2) Let *k*(*t*) = In⁡((2 − *t*)/*t*), *l*(*t*) = In⁡((2 − (1 − *t*))/(1 − *t*)), *k* ^−1^(*t*) = 2/(*e* ^*t*^ + 1), *l* ^−1^(*t*) = 1 − (2/(*e* ^*t*^ + 1)), and then Einstein *t*-conorms and *t*-norms are obtained as follows: *T*(*x*, *y*) = *xy*/(1 + (1 − *x*)(1 − *y*)), *S*(*x*, *y*) = (*x* + *y*)/(1 + *xy*).
(3) Let *k*(*t*) = In⁡((*γ* − (1 − *γ*)*t*)/*t*), *γ* > 0, *l*(*t*) = In⁡((*γ* − (1 − *γ*)(1 − *t*))/(1 − *t*)), *k* ^−1^(*t*) = *γ*/(*e* ^*t*^ + *γ* − 1), *l* ^−1^(*t*) = 1 − (*γ*/(*e* ^*t*^ + *γ* − 1)), and then Hamacher *t*-conorms and *t*-norms are obtained as follows:
  (5) $$\begin{array}{c} T\left(x,y\right)=\frac{xy}{\gamma +\left(1-\gamma \right)\left(x+y-xy\right)},\;\gamma >0,\\ S\left(x,y\right)=\frac{x+y-xy-\left(1-\gamma \right)xy}{1-\left(1-\gamma \right)xy},\;\gamma >0. \end{array}$$
  Based on the Archimedean *t*-conorms and *t*-norms, some operations of IVIFSs are discussed as follows.

**Definition 10**

Let $\tilde{\alpha }=\langle \left[a,b],[c,d\right]\rangle$, ${\tilde{\alpha }}_{1}=\langle \left[a_{1},b_{1}],[c_{1},d_{1}\right]\rangle$, ${\tilde{\alpha }}_{2}=\langle \left[a_{2},b_{2}],[c_{2},d_{2}\right]\rangle$ be three IVIFNs, *λ* ≥ 0, and then their operations could be defined as follows [19, 41–43]:

1. ${\tilde{\alpha }}^{\lambda }=\langle \left[k^{-1}(\lambda k(a)),k^{-1}(\lambda k(b))],[l^{-1}(\lambda l(c)\right),\;$ *l* ^−1^(*λl*(*d*))]〉;
2. $\lambda \tilde{\alpha }=\langle \left[l^{-1}(\lambda l(a)),l^{-1}(\lambda l(b))],[k^{-1}(\lambda k(c)\right)$, *k* ^−1^(*λk*(*d*))]〉, *λ* > 0;
3. ${\tilde{\alpha }}_{1}⊕{\tilde{\alpha }}_{2}=\langle \left[l^{-1}(l(a_{1})+l(a_{2})),l^{-1}(l(b_{1})+l(b_{2}))],[k^{-1}(k(c_{1})+k(c_{2})),k^{-1}(k(d_{1})+k(d_{2}))\right]\rangle$;
4. $\tilde{a}⊗\tilde{b}=\langle \left[k^{-1}(k(a_{1})+k(a_{2})),k^{-1}(k(b_{1})+k(b_{2}))],[l^{-1}(l(c_{1})+l(c_{2})),l^{-1}(l(d_{1})+l(d_{2}))\right]\rangle$.

Here, *l*(*t*) = *k*(1 − *t*), and *k* : [0,1] → [0, *∞*) is a strictly decreasing function.

### 2.3. HFSs

**Definition 11 (see [44])**

Let *X* be a universal set, and a HFS on *X* is in terms of a function that when applied to *X* will return a subset of [0,1], which can be represented as follows:

(6) $$\begin{array}{c} E=\left\{\langle x,h_{E}\left(x\right)\rangle \;|\;x\in X\right\}, \end{array}$$

where *h*_*E*_(*x*) is a set of values in [0, 1], denoting the possible membership degrees of the element *x* ∈ *X* to the set *E*. *h*_*E*_(*x*) is called a hesitant fuzzy element (HFE) [23], and *H* is the set of all HFEs. It is noteworthy that if *X* contains only one element, then *E* is called a hesitant fuzzy number (HFN), briefly denoted by *E* = {*h*_*E*_(*x*)}. The set of all hesitant fuzzy numbers is represented as HFNS.

Torra [44] defined some operations on HFNs, and Xia and Xu [19, 22] defined some new operations on HFNs and the score function.

**Definition 12 (see [43])**

Let *h*, *h*_1_, and *h*_2_ be three HFNs, *λ* ≥ 0, and then four operations are defined as follows:

1. *h* ^*λ*^ = ⋃_*γ*∈*h*_{*k* ^−1^(*λk*(*γ*))};
2. *λh* = ⋃_*γ*∈*h*_{*l* ^−1^(*λl*(*γ*))};
3. *h*_1_ ⊕ *h*_2_ = ⋃_*γ*_1_∈*h*_1_,*γ*_2_∈*h*_2__{*l* ^−1^(*l*(*γ*_1_) + *l*(*γ*_2_))};
4. *h*_1_ ⊗ *h*_2_ = ⋃_*γ*_1_∈*h*_1_,*γ*_2_∈*h*_2__{*k* ^−1^(*k*(*γ*_1_) + *k*(*γ*_2_))}.

Here, *l*(*t*) = *k*(1 − *t*), and *k* : [0,1]→[0, *∞*) is a strictly decreasing function.

**Definition 13 (see [19])**

Let *h* ∈ HFNs, and *s*(*h*) = (1/#*h*)∑_*γ*∈*h*_ *γ* is called the score function of *h*, where #*h* is the number of elements in *h*. For two HFNs *h*_1_ and *h*_2_, if *s*(*h*_1_) > *s*(*h*_2_), then *h*_1_ > *h*_2_; if *s*(*h*_1_) = *s*(*h*_2_), then *h*_1_ = *h*_2_.

**Example 14**

Let *h*_1_ = {0.3,0.5,0.6}, *h*_2_ = {0.4,0.7} be two HFNs. According to Definition 13, *s*(*h*_1_) = (1/3) × (0.3 + 0.5 + 0.6) = 0.4667, *s*(*h*_2_) = 0.55, *s*(*h*_2_) > *s*(*h*_1_), so *h*_2_ > *h*_1_.

Furthermore, Torra and Narukawa [18, 44] proposed an aggregation principle for HFEs.

**Definition 15 (see [18, 44])**

Let *E* = {*h*_1_, *h*_2_,…, *h*_*n*_} be a set of *n* HFEs, let *ϑ* be a function on *E*, and let *ϑ* : [0,1]^*n*^ → [0,1], and then

(7) $$\begin{array}{c} \vartheta_{E}=\underset{\gamma \in h_{1}\times h_{2}\times \cdots \times h_{n}}{⋃}\left\{\vartheta \left(\gamma \right)\right\}. \end{array}$$

## 3. HIVIFSs and Their Operations

HFSs are the extension of traditional fuzzy sets, and their membership degree of an element is a set of several possible values between 0 and 1. In some cases, decision-makers usually cannot estimate criteria values of alternatives with an exact numerical value when the information is not precisely known. Therefore, interval values in fuzzy sets can represent it better than specific numbers, such as IVFSs and IVIFSs. Furthermore, IVIFSs could describe the object being “neither this nor that,” and the membership degree and nonmembership degree of IVIFSs are interval values, respectively. Thus, precise numerical values in HFSs can be replaced by IVIFSs, which are more flexible in the real world, and this is what this section will solve.

**Definition 16**

Assume that *X* is a finite universal set. A HIVIFS *A* in *X* is an object in the following form:

(8) $$\begin{array}{c} E=\left\{\langle x,H_{E}\left(x\right)\rangle \;|\;x\in X\right\}, \end{array}$$

where *H*_*E*_(*x*) is a finite set of values in IVIFSs, denoting the possible membership degrees and nonmembership degrees of the element *x* ∈ *X* to the set *E*.

Based on the definition given above,

(9) $$\begin{array}{c} H_{E}\left(x\right)=\left\{\overset{n(H_{E}(x))}{\underset{i=1}{⋃}}\langle \left[\mu_{E_{i}}^{L}\left(x\right),\mu_{E_{i}}^{U}\left(x\right)\right],\left[\nu_{E_{i}}^{L}\left(x\right),\nu_{E_{i}}^{U}\left(x\right)\right]\rangle \right\}, \end{array}$$

where 0 ≤ *μ*_*E*_1__^*L*^(*x*) ≤ *μ*_*E*_1__^*U*^(*x*) ≤ *μ*_*E*_2__^*L*^(*x*) ≤ *μ*_*E*_2__^*U*^(*x*)≤⋯*μ*_*n*(*H*_*E*_(*x*))_^*L*^(*x*) ≤ *μ*_*n*(*H*_*E*_(*x*))_^*U*^(*x*) ≤ 1, 0 ≤ *μ*_*E*_*i*__^*U*^(*x*) + *ν*_*E*_*i*__^*U*^(*x*) ≤ 1, *μ*_*E*_*i*__^*L*^(*x*) ≥ 0, *ν*_*E*_*i*__^*L*^(*x*) ≥ 0, and *n*(*H*_*E*_(*x*)) ≥ 1. Actually, HIVIFSs have several possible membership degrees taking the form of IVIFSs instead of FSs in HFSs. If *n*(*H*_*E*_(*x*)) = 1, then the HIVIFS is reduced to an IVIFS; if *μ*_*E*_*i*__^*L*^(*x*) = *μ*_*E*_*i*__^*U*^(*x*)  (*i* = 1,2,…, *n*(*H*_*E*_(*x*))) and *ν*_*E*_*i*__^*L*^(*x*) = *ν*_*E*_*i*__^*U*^(*x*) = 0  (*i* = 1,2,…, *n*(*H*_*E*_(*x*))), then the HIVIFS is reduced to a HFS; if *μ*_*E*_*i*__^*L*^(*x*) = *μ*_*E*_*i*__^*U*^(*x*)  (*i* = 1,2,…, *n*(*H*_*E*_(*x*))) or *ν*_*E*_*i*__^*L*^(*x*) = *ν*_*E*_*i*__^*U*^(*x*)  (*i* = 1,2,…, *n*(*H*_*E*_(*x*))), then the HIVIFS is reduced to a HIVFS; if *μ*_*E*_*i*__^*L*^(*x*) = *μ*_*E*_*i*__^*U*^(*x*)  (*i* = 1,2,…, *n*(*H*_*E*_(*x*))) and *ν*_*E*_*i*__^*L*^(*x*) = *ν*_*E*_*i*__^*U*^(*x*)  (*i* = 1,2,…, *n*(*H*_*E*_(*x*))), then the HIVIFS is reduced to a HIFS. Furthermore, *H*_*E*_(*x*) is called a hesitant interval-valued intuitionistic fuzzy element (HIVIFE), and *E* is the set of all HIVIFEs. In particular, if *X* has only one element, 〈*x*, *H*_*E*_(*x*)〉 is called a hesitant interval-valued intuitionistic fuzzy number (HIVIFN), briefly denoted by

(10) $$\begin{array}{c} H_{E}=\left\{\overset{n(H_{E})}{\underset{i=1}{⋃}}\langle \left[a_{i},b_{i}\right],\left[c_{i},d_{i}\right]\rangle \right\}. \end{array}$$

The set of all HIVIFNs is denoted by HIVIFNS.

**Definition 17**

Let *A* ∈ HIVIFS(*X*), *A* = {〈*x*, *H*_*A*_(*x*)〉 | *x* ∈ *X*}, and for all *x* ∈ *X*, Π_*A*_(*x*) = ⋃_*i*=1_^*n*(*H*_*A*_(*x*))^{[1 − *μ*_*A*_*i*__^*U*^(*x*) − *ν*_*A*_*i*__^*U*^(*x*), 1 − *μ*_*A*_*i*__^*L*^(*x*) − *ν*_*A*_*i*__^*L*^(*x*)]}. Then, Π_*A*_(*x*) is called the hesitant interval-valued intuitionistic index of *x*.

**Example 18**

Let *X* = {*x*_1_, *x*_2_}, and let *A* = {〈*x*_1_, {〈[0.3,0.4], [0.1,0.2]〉, 〈0.4,0.2〉}〉, 〈*x*_2_, {〈[0.5,0.6],[0.2,0.4]〉}〉} be a HIVIFS, and then Π_*A*_(*x*_1_) = {[0.4,0.6], 0.4}, Π_*A*_(*x*_2_) = {[0,0.3]}. Thus, Π_*A*_(*x*) = {〈*x*_1_, {[0.4,0.6], 0.4}〉, 〈*x*_2_, {[0,0.3]}〉}.

The operations of HIVIFNs are defined as follows.

**Definition 19**

Let *H*_1_ = {⋃_*i*_1_=1_^*n*(*H*_1_)^〈[*a*_*i*_1__, *b*_*i*_1__], [*c*_*i*_1__, *d*_*i*_1__]〉} and *H*_2_ = {⋃_*i*_2_=1_^*n*(*H*_2_)^〈[*a*_*i*_2__, *b*_*i*_2__], [*c*_*i*_2__, *d*_*i*_2__]〉} be two HIVIFNs, *λ* ≥ 0, and four operations are defined as follows:

1. *λH*_1_ = ⋃_*i*_1_=1_^*n*(*H*_1_)^{〈[*l* ^−1^(*λl*(*a*_*i*_1__)), *l* ^−1^(*λl*(*b*_*i*_1__))], [*k* ^−1^(*λk*(*c*_*i*_1__)), *k* ^−1^(*λk*(*d*_*i*_1__))]〉};
2. (*H*_1_)^*λ*^ = ⋃_*i*=1_^*n*(*H*_1_)^{〈[*k* ^−1^(*λk*(*a*_*i*_1__)), *k* ^−1^(*λk*(*b*_*i*_1__))], [*l* ^−1^(*λl*(*c*_*i*_1__)), *l* ^−1^(*λl*(*d*_*i*_1__))]〉};
3. *H*_1_ ⊕ *H*_2_ = ⋃_*i*_1_=1_^*n*(*H*_1_)^⋃_*i*_2_=1_^*n*(*H*_2_)^{〈[*l* ^−1^(*l*(*a*_*i*_1__) + *l*(*a*_*i*_2__)), *l* ^−1^(*l*(*b*_*i*_1__) + *l*(*b*_*i*_2__))], [*k* ^−1^(*k*(*c*_*i*_1__) + *k*(*c*_*i*_2__)), *k* ^−1^(*k*(*d*_*i*_1__)+*k*(*d*_*i*_2__))]〉};
4. *H*_1_ ⊗ *H*_2_ = ⋃_*i*_1_=1_^*n*(*H*_1_)^⋃_*i*_2_=1_^*n*(*H*_2_)^{〈[*k* ^−1^(*k*(*a*_*i*_1__) + *k*(*a*_*i*_2__)), *k* ^−1^(*k*(*b*_*i*_1__) + *k*(*b*_*i*_2__))], [*l* ^−1^(*l*(*c*_*i*_1__) + *l*(*c*_*i*_2__)), *l* ^−1^(*l*(*d*_*i*_1__)+*l*(*d*_*i*_2__))]〉}.

Here, *l*(*t*) = *k*(1 − *t*), and *k* : [0,1]→[0, *∞*) is a strictly decreasing function.

**Example 20**

Let *H*_1_ = {〈[0.1,0.3], [0.2,0.4]〉, 〈[0.2,0.3],  [0.3,0.4]〉} and *H*_2_ = {〈[0.3,0.4], [0.2,0.3]〉} be two HIVIFNs, and *k*(*x*) = −In⁡*x*, *k* ^−1^(*x*) = *e* ^−*x*^,  *l*(*x*) = −In⁡(1 − *x*), *l* ^−1^(*x*) = 1 − *e* ^−*x*^, and *λ* = 2. The following can be calculated:

1. 2*H*_1_ = {〈[1 − *e* ^−2(−log⁡(1−0.1))^, 1 − *e* ^−2(−log⁡(1−0.3))^], [*e* ^−2(−log⁡0.2)^, *e* ^−2(−log⁡0.4)^]〉, 〈[1 − *e* ^−2(−log⁡(1−0.2))^,1 − *e* ^−2(−log⁡(1−0.3))^], [*e* ^−2(−log⁡0.3)^, *e* ^−2(−log⁡0.4)^]〉} = {〈[0.19,0.51], [0.04,0.16]〉,〈[0.36,0.51], [0.09,0.16]〉};
2. (*H*_1_)^2^ = {〈[0.01,0.09], [0.36,0.64]〉, 〈[0.04,0.09],[0.51,0.64]〉};
3. *H*_1_ ⊕ *H*_2_ = {〈[0.37,0.58], [0.04,0.12]〉, 〈[0.44,0.58],[0.06,0.12]〉};
4. *H*_1_ ⊗ *H*_2_ = {〈[0.03,0.12], [0.36,0.58]〉, 〈[0.06,0.12],[0.44,0.58]〉}.

**Theorem 21**

Let *H*_1_, *H*_2_, *H*_3_ ∈ *HIVIFNS*, *λ*, *λ*_1_, *λ*_2_ > 0, and then

1. *H*_1_ ⊕ *H*_2_ = *H*_2_ ⊕ *H*_1_;
2. *H*_1_ ⊗ *H*_2_ = *H*_2_ ⊗ *H*_1_;
3. *λH*_1_ ⊕ *λH*_2_ = *λ*(*H*_1_ ⊕ *H*_2_);
4. (*H*_1_)^*λ*^ ⊗ (*H*_2_)^*λ*^ = (*H*_1_ ⊗ *H*_2_)^*λ*^;
5. (*H*_1_ ⊕ *H*_2_) ⊕ *H*_3_ = *H*_1_ ⊕ (*H*_2_ ⊕ *H*_3_);
6. (*H*_1_ ⊗ *H*_2_) ⊗ *H*_3_ = *H*_1_ ⊗ (*H*_2_ ⊗ *H*_3_);
7. ((*H*_1_)^*λ*_1_^)^*λ*_2_^ = (*H*_1_)^*λ*_1_*λ*_2_^.

**Proof**

According to Definition 19, it is clear that (1), (2), (5), and (6) are obvious. (3), (4), and (7) will be proved as follows:

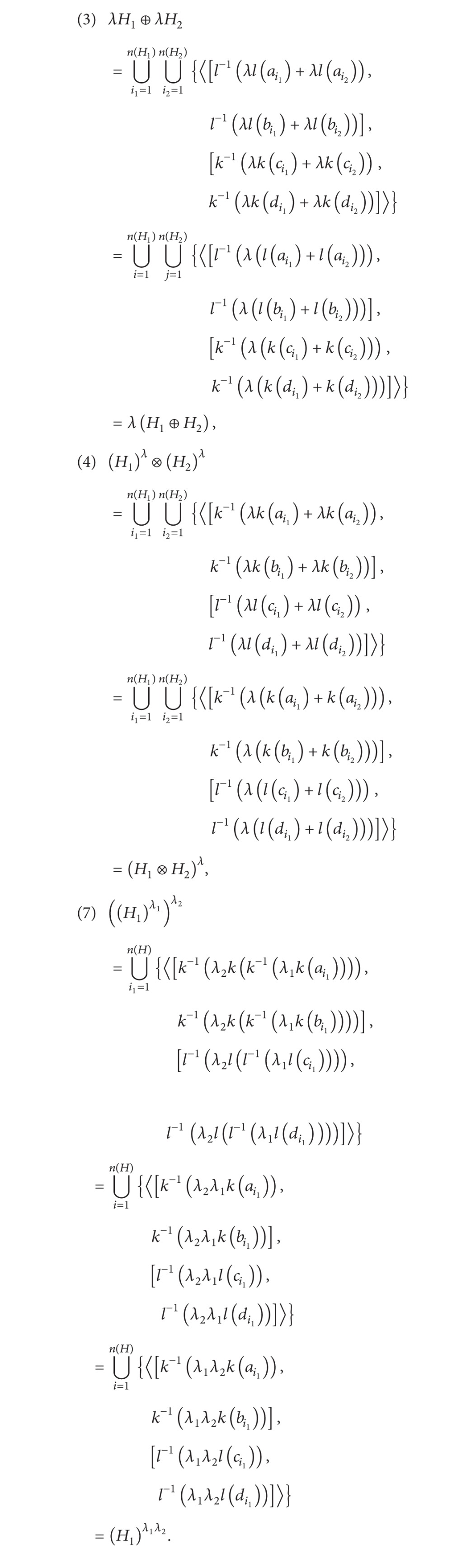
(11)
The proof is completed.

Based on Definitions 5, 6, and 13, the ranking method for HIVIFNs is defined as follows.

**Definition 22**

Let *H* ∈ HIVIFNs, $\tilde{S}\left(H\right)=\left(1/\#H\right)\sum_{\gamma \in H}\gamma$ is called the score function of *H*, where #*H* is the number of the interval-valued intuitionistic fuzzy values in *H*. For two HIVIFNs *H*_1_ and *H*_2_, if $\tilde{S}(H_{1})>\tilde{S}(H_{2})$, then *H*_1_ > *H*_2_; if $\tilde{S}(H_{1})=\tilde{S}(H_{2})$, then *H*_1_ = *H*_2_.

Note that $\tilde{S}(H_{1})$ and $\tilde{S}(H_{2})$ could be compared by utilizing Definitions 5 and 6.

**Example 23**

Let *H*_1_ = {〈[0.3,0.4], [0.1,0.2]〉, 〈[0.3,0.5],  [0.2,0.4]〉} and *H*_2_ = {〈[0.3,0.4], [0.2,0.3]〉} be two HIVIFNs, and then

(12) S~(H1)=12×〈[0.3+0.3,0.4+0.5],[0.1+0.2,0.2+0.4]〉=〈[0.30,0.45],[0.15,0.30]〉,S~(H2)=〈[0.3,0.4],[0.2,0.3]〉.

According to Definitions 5 and 6,

(13) L(S~(H1)) =0.30+0.45−0.30×(1−0.45)−0.15×(1−0.30)2 =0.24,L(S~(H2)) =0.3+0.4−0.3×(1−0.4)−0.2×(1−0.3)2 =0.19.

Hence, $\tilde{S}(H_{1})>\tilde{S}(H_{2})$, which indicates that *H*_1_ is preferred to *H*_2_.

## 4. HIVIFN Aggregation Operators and Their Applications in MCDM Problems

In this section, HIVIFN aggregation operators are proposed, and some properties of these operators are discussed. In particular, some hesitant interval-valued intuitionistic fuzzy algebraic aggregation operators are proposed based on algebraic *t*-conorms and *t*-norms. Then, how to utilize these operators to MCDM problems is discussed as well.

### 4.1. HIVIFN Aggregation Operators

**Definition 24**

Let *H*_*j*_ (*j* = 1,2,…, *n*) be a collection of HIVIFNs, and HIVIFNWA: HIVIFNS^*n*^ → HIVIFNS, and then

(14) $$\begin{array}{c} \text{HIVIFNW}{\text{A}}_{w}\left(H_{1},H_{2},\ldots ,H_{n}\right)=\overset{n}{\underset{j=1}{⨁}}w_{j}H_{j}. \end{array}$$

The HIVIFNWA operator is called the HIVIFN weighted averaging operator of dimension *n*, where *w* = (*w*_1_, *w*_2_,…, *w*_*n*_) is the weight vector of *H*_*j*_  (*j* = 1,2,…, *n*), with *w*_*j*_ ≥ 0  (*j* = 1,2,…, *n*) and ∑_*j*=1_^*n*^ *w*_*j*_ = 1.

**Theorem 25**

Let *H*_*j*_ = {⋃_*i*_*j*_=1_^*n*(*H*_*j*_)^〈[*a*_*i*_*j*__, *b*_*i*_*j*__], [*c*_*i*_*j*__, *d*_*i*_*j*__]〉}  (*j* = 1,2,…, *n*) be a collection of HIVIFNs and let *w* = (*w*_1_, *w*_2_,…, *w*_*n*_) be the weight vector of *H*_*j*_  (*j* = 1,2,…, *n*), with *w*_*j*_ ≥ 0  (*j* = 1,2,…, *n*) and ∑_*j*=1_^*n*^ *w*_*j*_ = 1. Then, the aggregated result using the HIVIFNWA operator is also a HIVIFN, and

(15) HIVIFNWAw(H1,H2,…,Hn)=⋃i1=1n(H1)⋯⋃in=1n(Hn){〈[l−1(∑j=1nwjl(aij)),  l−1(∑j=1nwjl(bij))],[k−1(∑j=1nwjk(cij)),k−1(∑j=1nwjk(dij))]〉}.

**Proof**

By using mathematical induction on *n*, we have the following.

(1) For *n* = 2, since
  (16) w1H1=⋃i1=1n(H1){〈[l−1(w1l(ai1)),l−1(w1l(bi1))], [k−1(w1k(ci1)),k−1(w1k(di1))]〉},w2H2=⋃i2=1n(H2){〈[l−1(w2l(ai2)),l−1(w2l(bi2))], [k−1(w2k(ci2)),k−1(w2k(di2))]〉},

the following can be obtained:

(17) HIVIFNWAw(H1,H2) =w1H1⊕w2H2 =⋃i1=1n(H1) ⋃i2=1n(H2){〈[l−1(w1l(ai1)+w2l(ai2)), l−1(w1l(bi1)+w2l(bi2))], [k−1(w1k(ci1)+w2k(ci2)), k−1(w1k(di1)+w2k(di2))]〉}.

(2) If (15) holds for *n* = *k*, then

(18) HIVIFNWAw(H1,H2,…,Hk)=⋃i1=1n(H1)⋯⋃ik=1n(Hk){〈[l−1(w1l(ai1)+w2l(ai2)+⋯wkl(aik)), l−1(w1l(bi1)+w2l(bi2)+⋯+wkl(bik))],[k−1(w1k(ci1)+w2k(ci2)+⋯wkk(cik)),k−1(w1k(di1)+w2k(di2)+⋯wkk(dik))]〉}=⋃i1=1n(H1)⋯⋃ik=1n(Hk){〈[l−1(∑j=1kwjl(aij)),l−1(∑j=1kwjl(bij))],[k−1(∑j=1kwjk(cij)),k−1(∑j=1kwjk(dij))]〉}.

When *n* = *k* + 1, in terms of (1) and (3) in Definition 19,

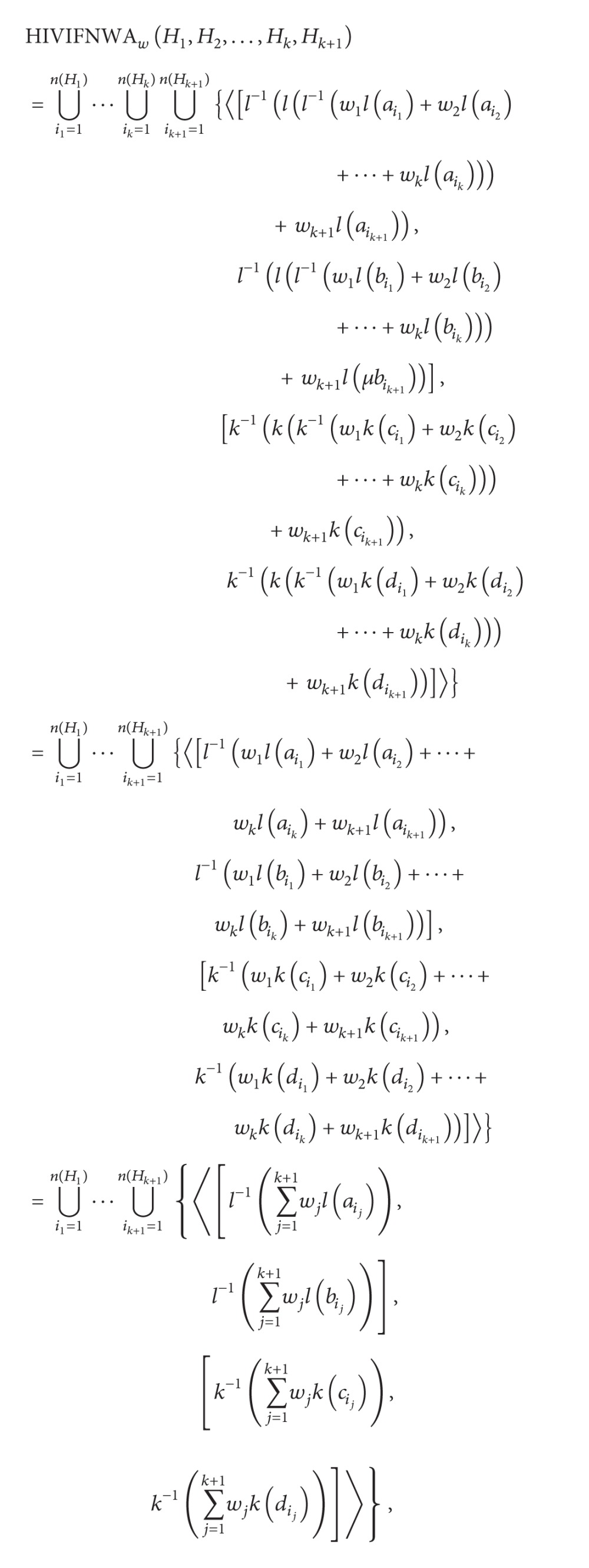
(19)
that is, (15) holds for *n* = *k* + 1; thus, (15) holds for all *n*. Then,

(20) HIVIFNWAw(H1,H2,…,Hn)=⋃i1=1n(H1)⋯⋃in=1n(Hn){〈[l−1(w1l(ai1)+w2l(ai2)+⋯wnl(ain)),l−1(w1l(bi1)+w2l(bi2)+⋯+wnl(bin))],[k−1(w1k(ci1)+w2k(ci2)+⋯wnk(cin)),k−1(w1k(di1)+w2k(di2)+⋯wnk(din))]〉}=⋃i1=1n(H1)⋯⋃in=1n(Hn){〈[l−1(∑j=1nwjl(aij)),l−1(∑j=1nwjl(bij))],[k−1(∑j=1nwjk(cij)),k−1(∑j=1nwjk(dij))]〉}.

**Definition 26**

Let *H*_*j*_  (*j* = 1,2,…, *n*) be a collection of HIVIFNs, HIVIFNWG: HIVIFNS^*n*^ → HIVIFNS, and then

(21) $$\begin{array}{c} \text{HIVIFNW}{\text{G}}_{w}\left(H_{1},H_{2},\ldots ,H_{n}\right)=\overset{n}{\underset{j=1}{⨂}}{\left(H_{j}\right)}^{w_{j}}. \end{array}$$

The HIVIFNWG operator is called the HIVIFN weighted geometric operator of dimension *n*, and *w* = (*w*_1_, *w*_2_,…, *w*_*n*_) is the weight vector of *H*_*j*_  (*j* = 1,2,…, *n*), with *w*_*j*_ ≥ 0  (*j* = 1,2,…, *n*) and ∑_*j*=1_^*n*^ *w*_*j*_ = 1.

Similarly, the following theorems can be obtained.

**Theorem 27**

Let *H*_*j*_ = {⋃_*i*_*j*_=1_^*n*(*H*_*j*_)^〈[*a*_*i*_*j*__, *b*_*i*_*j*__], [*c*_*i*_*j*__, *d*_*i*_*j*__]〉}  (*j* = 1,2,…, *n*) be a collection of HIVIFNs and let *w* = (*w*_1_, *w*_2_,…, *w*_*n*_) be the weight vector of *A*_*j*_  (*j* = 1,2,…, *n*), with *w*_*j*_ ≥ 0  (*j* = 1,2,…, *n*) and ∑_*j*=1_^*n*^ *w*_*j*_ = 1. Then, the aggregated result using the HIVIFNWG operator is also a HIVIFN, and

(22) HIVIFNWGw(H1,H2,…,Hn)=⋃i1=1n(H1)⋯⋃ik=1n(Hn){〈[k−1(∑j=1nwjk(aij)),  k−1(∑j=1nwjk(bij))],[l−1(∑j=1nwjl(cij)),l−1(∑j=1nwjl(dij))]〉}.

**Definition 28**

Let *H*_*j*_  (*j* = 1,2,…, *n*) be a collection of HIVIFNs, HIVIFNWAA: HIVIFNS^*n*^ → HIVIFNS, and then

(23) $$\begin{array}{c} \text{HIVIFNWA}{\text{A}}_{w}\left(H_{1},H_{2},\ldots ,H_{n}\right)={\left(\overset{n}{\underset{j=1}{⨁}}w_{j}{\left(H_{j}\right)}^{2}\right)}^{1/2}. \end{array}$$

The HIVIFNWAA operator is called the HIVIFN weighted arithmetic averaging operator of dimension *n*, where *w* = (*w*_1_, *w*_2_,…, *w*_*n*_) is the weight vector of *H*_*j*_  (*j* = 1,2,…, *n*), with *w*_*j*_ > 0  (*j* = 1,2,…, *n*) and ∑_*j*=1_^*n*^ *w*_*j*_ = 1.

**Theorem 29**

Let *H*_*j*_ = {⋃_*i*_*j*_=1_^*n*(*H*_*j*_)^〈[*a*_*i*_*j*__, *b*_*i*_*j*__], [*c*_*i*_*j*__, *d*_*i*_*j*__]〉}  (*j* = 1,2,…, *n*) be a collection of HIVIFNs and let *w* = (*w*_1_, *w*_2_,…, *w*_*n*_) be the weight vector of *H*_*j*_  (*j* = 1,2,…, *n*), with *w*_*j*_ ≥ 0  (*j* = 1,2,…, *n*) and ∑_*j*=1_^*n*^ *w*_*j*_ = 1. Then, the aggregated result using the HIVIFNWAA operator is also a HIVIFN, and
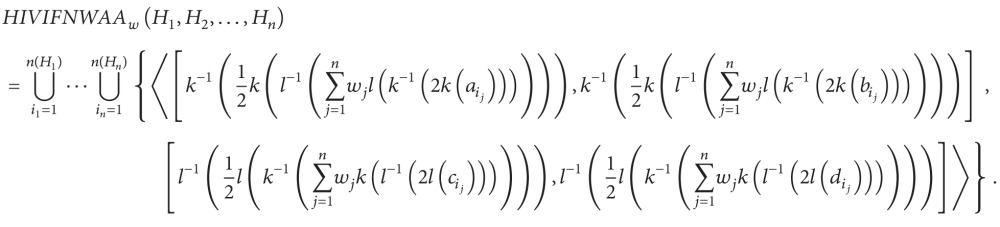
(24)

**Definition 30**

Let *H*_*j*_ (*j* = 1,2,…, *n*) be a collection of HIVIFNs, HIVIFNWAG: HIVIFNS^*n*^ → HIVIFNS, and then

(25) $$\begin{array}{c} \text{HIVIFNWA}{\text{G}}_{w}\left(H_{1},H_{2},\ldots ,H_{n}\right)=\frac{1}{2}\left(\overset{n}{\underset{j=1}{⨂}}{\left({2H}_{j}\right)}^{w_{j}}\right). \end{array}$$

The HIVIFNWAG operator is called the HIVIFN weighted arithmetic geometric operator of dimension *n*, where *w* = (*w*_1_, *w*_2_,…, *w*_*n*_) is the weight vector of *H*_*j*_  (*j* = 1,2,…, *n*), with *w*_*j*_ > 0  (*j* = 1,2,…, *n*) and ∑_*j*=1_^*n*^ *w*_*j*_ = 1.

**Theorem 31**

Let *H*_*j*_ = {⋃_*i*_*j*_=1_^*n*(*H*_*j*_)^〈[*a*_*i*_*j*__, *b*_*i*_*j*__], [*c*_*i*_*j*__, *d*_*i*_*j*__]〉}  (*j* = 1,2,…, *n*) be a collection of HIVIFNs and let *w* = (*w*_1_, *w*_2_,…, *w*_*n*_) be the weight vector of *A*_*j*_  (*j* = 1,2,…, *n*), with *w*_*j*_ ≥ 0  (*j* = 1,2,…, *n*) and ∑_*j*=1_^*n*^ *w*_*j*_ = 1. Then, the aggregated result using the HIVIFNWAG operator is also a HIVIFN, and
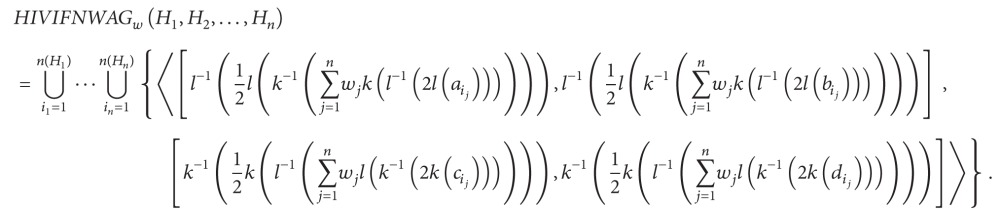
(26)

**Definition 32**

Let *H*_*j*_  (*j* = 1,2,…, *n*) be a collection of HIVIFNs, GHIVIFNWA: HIVIFNS^*n*^ → HIVIFNS, and then

(27) $$\begin{array}{c} \text{GHIVIFNW}{\text{A}}_{w}\left(H_{1},H_{2},\ldots ,H_{n}\right)={\left(\overset{n}{\underset{j=1}{⨁}}w_{j}{\left(H_{j}\right)}^{\lambda }\right)}^{1/\lambda }. \end{array}$$

The GHIVIFNWA operator is called the generalized HIVIFN weighted averaging operator of dimension *n*, where *w* = (*w*_1_, *w*_2_,…, *w*_*n*_) is the weight vector of *H*_*j*_  (*j* = 1,2,…, *n*), with *w*_*j*_ > 0   (*j* = 1,2,…, *n*) and ∑_*j*=1_^*n*^ *w*_*j*_ = 1. If *λ* = 1, the GHIVIFNWA operator is reduced to the HIVIFNWA operator. If *λ* = 2, the GHIVIFNWA operator is reduced to the HIVIFNWAA operator.

**Theorem 33**

Let *H*_*j*_ = {⋃_*i*_*j*_=1_^*n*(*H*_*j*_)^〈[*a*_*i*_*j*__, *b*_*i*_*j*__], [*c*_*i*_*j*__, *d*_*i*_*j*__]〉}  (*j* = 1,2,…, *n*) be a collection of HIVIFNs and let *w* = (*w*_1_, *w*_2_,…, *w*_*n*_) be the weight vector of *H*_*j*_  (*j* = 1,2,…, *n*), with *λ* > 0, *w*_*j*_ ≥ 0  (*j* = 1,2,…, *n*), and ∑_*j*=1_^*n*^ *w*_*j*_ = 1. Then, the aggregated result using the GHIVIFNWA operator is also a HIVIFN, and
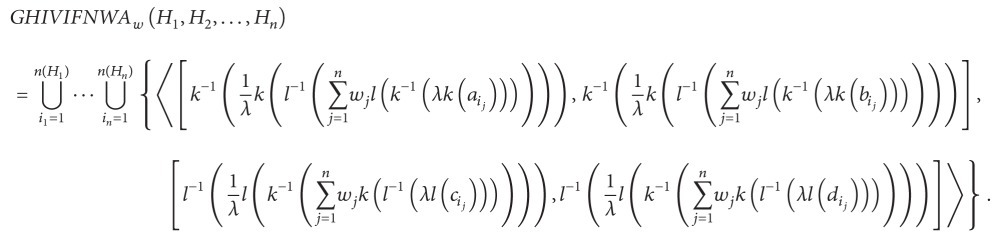
(28)

**Definition 34**

Let *H*_*j*_ (*j* = 1,2,…, *n*) be a collection of HIVIFNs, GHIVIFNWG: HIVIFNS^*n*^ → HIVIFNS, and then

(29) $$\begin{array}{c} \text{GHIVIFNW}{\text{G}}_{w}\left(H_{1},H_{2},\ldots ,H_{n}\right)=\frac{1}{\lambda }\left(\overset{n}{\underset{j=1}{⨂}}{\left({\lambda H}_{j}\right)}^{w_{j}}\right). \end{array}$$

The GHIVIFNWG operator is called the generalized HIVIFN weighted geometric operator of dimension *n*, where *w* = (*w*_1_, *w*_2_,…, *w*_*n*_) is the weight vector of *H*_*j*_  (*j* = 1,2,…, *n*), with *w*_*j*_ > 0  (*j* = 1,2,…, *n*) and ∑_*j*=1_^*n*^ *w*_*j*_ = 1. If *λ* = 1, the GHIVIFNWG operator is reduced to the HIVIFNWG operator. If *λ* = 2, then the GHIVIFNWG operator is reduced to the HIVIFNWAG operator.

**Theorem 35**

Let *H*_*j*_ = {⋃_*i*_*j*_=1_^*n*(*H*_*j*_)^〈[*a*_*i*_*j*__, *b*_*i*_*j*__], [*c*_*i*_*j*__, *d*_*i*_*j*__]〉}  (*j* = 1,2,…, *n*) be a collection of HIVIFNs and let *w* = (*w*_1_, *w*_2_,…, *w*_*n*_) be the weight vector of *A*_*j*_  (*j* = 1,2,…, *n*), with *λ* > 0, *w*_*j*_ ≥ 0  (*j* = 1,2,…, *n*), and ∑_*j*=1_^*n*^ *w*_*j*_ = 1. Then, the aggregated result using the GHIVIFNWG operator is also a HIVIFN, and
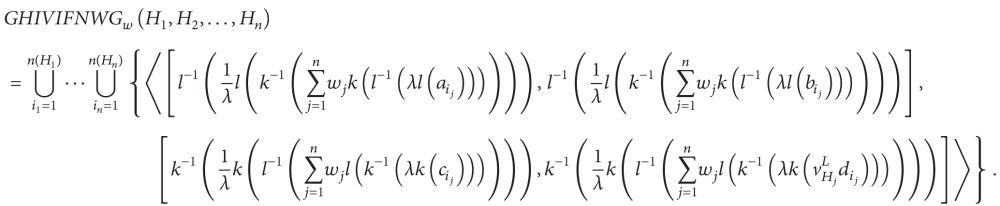
(30)

Note that Theorems 27–35 could be proved by using the mathematical induction method and are omitted here.

Based on these hesitant interval-valued intuitionistic fuzzy aggregation operators, it is easy to obtain the following properties.

**Property 1 (idempotency)**

Let *H*_*j*_ = {⋃_*i*_*j*_=1_^*n*(*H*_*j*_)^〈[*a*_*i*_*j*__, *b*_*i*_*j*__], [*c*_*i*_*j*__, *d*_*i*_*j*__]〉}  (*j* = 1,2,…, *n*) be a collection of HIVIFNs. If *H*_*j*_ = *H* = {⋃_*i*=1_^*n*(*H*)^〈[*a*_*i*_, *b*_*i*_], [*c*_*i*_, *d*_*i*_]〉} for all *i* = 1,2,…, *n*, then

(31) $$\begin{array}{c} \text{GHIVIFNWA}\left(H_{1},H_{2},\ldots ,H_{n}\right)=H,\\ \text{GHIVIFNWG}\left(H_{1},H_{2},\ldots ,H_{n}\right)=H. \end{array}$$

**Proof**

According to Theorem 33 and *H*_*j*_ = *H* = {⋃_*i*=1_^*n*(*H*)^〈[*a*_*i*_, *b*_*i*_], [*c*_*i*_, *d*_*i*_]〉} for all *i* = 1,2,…, *n*,
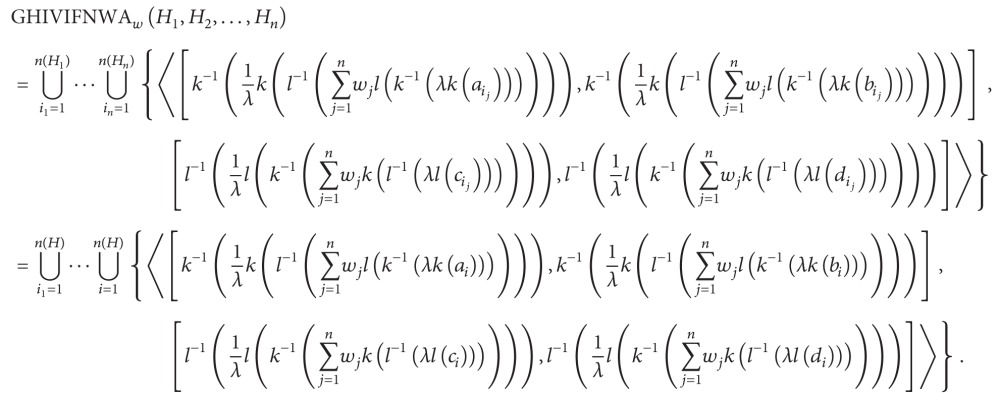
(32)
Since ∑_*j*=1_^*n*^ *w*_*j*_ = 1,

(33) k−1(1λk(l−1(∑j=1nwjl(k−1(λk(ai))))))=k−1(1λk(l−1(l(k−1(λk(ai))))))=k−1(1λk(k−1(λk(ai))))=k−1(1λ(λk(ai)))=k−1(1λλk(ai))=k−1(k(ai))=ai,k−1(1λk(l−1(∑j=1nwjl(k−1(λk(bi))))))=bi,l−1(1λl(k−1(∑j=1nwjk(l−1(λl(ci))))))=ci,l−1(1λl(k−1(∑j=1nwjk(l−1(λl(di))))))=di.

Hence, GHIVIFNWA_*w*_(*H*_1_, *H*_2_,…, *H*_*n*_) = ⋃_*i*=1_^*n*(*H*)^{〈[*a*_*i*_, *b*_*i*_], [*c*_*i*_, *d*_*i*_]〉} = *H*.

Similarly, GHIVIFNWG(*H*_1_, *H*_2_,…, *H*_*n*_) = *H*.

**Property 2 (commutativity)**

Let *H*_*j*_ = {⋃_*i*_*j*_=1_^*n*(*H*_*j*_)^〈[*a*_*i*_*j*__, *b*_*i*_*j*__], [*c*_*i*_*j*__, *d*_*i*_*j*__]〉}  (*j* = 1,2,…, *n*) be a collection of HIVIFNs and let ${\tilde{H}}_{j}=\left\{{⋃}_{i_{j}=1}^{n({\tilde{H}}_{j})}\langle \left[{\tilde{a}}_{i_{j}},{\tilde{b}}_{i_{j}}\right],\left[{\tilde{c}}_{i_{j}},{\tilde{d}}_{i_{j}}\right]\rangle \right\}\;\;\left(j=1,2,\ldots ,n\right)$ be any permutation of *H*_*j*_, and then

(34) GHIVIFNWA(H~1,H~2,…,H~n)  =GHIVIFNWA(H1,H2,…,Hn),GHIVIFNWG(H~1,H~2,…,H~n)  =GHIVIFNWG(H1,H2,…,Hn).

**Proof**

Since ${\tilde{H}}_{j}=\left\{{⋃}_{i_{j}=1}^{n({\tilde{H}}_{j})}\langle \left[{\tilde{a}}_{i_{j}},{\tilde{b}}_{i_{j}}\right],\left[{\tilde{c}}_{i_{j}},{\tilde{d}}_{i_{j}}\right]\rangle \right\}\;\;\left(j=1,2,\ldots ,n\right)$ is any permutation of *H*_*j*_,
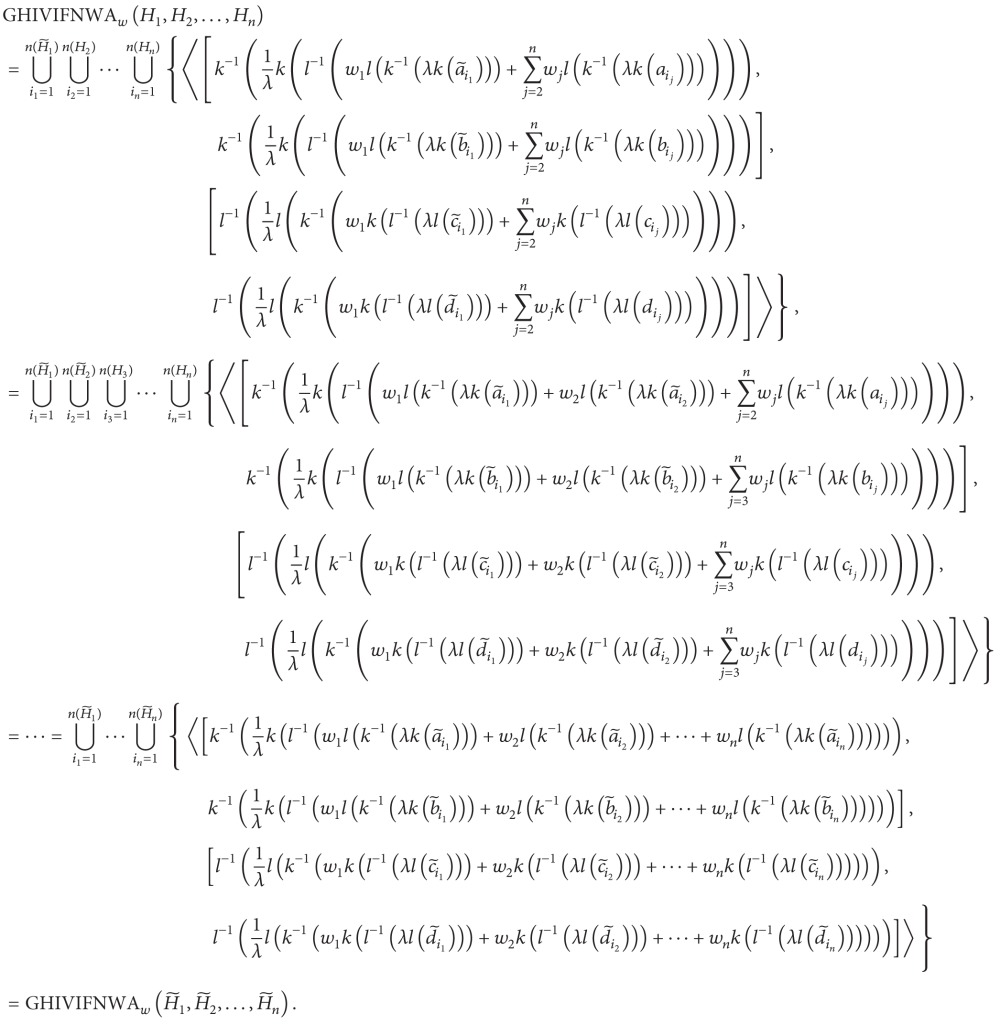
(35)

Similarly, $\text{GHIVIFNWG}({\tilde{H}}_{1},{\tilde{H}}_{2},\ldots ,{\tilde{H}}_{n})=\text{GHIVIFNWG}(H_{1},H_{2},\ldots ,H_{n})$.

**Property 3 (boundary)**

Let *H*_*j*_ = {⋃_*i*_*j*_=1_^*n*(*H*_*j*_)^〈[*a*_*i*_*j*__, *b*_*i*_*j*__], [*c*_*i*_*j*__, *d*_*i*_*j*__]〉}  (*j* = 1,2,…, *n*) be a collection of HIVIFNs, and then

(36) $$\begin{array}{c} H^{-}\leq \text{GHIVIFAWA}\left(H_{1},H_{2},\ldots ,H_{n}\right)\leq H^{+},\\ H^{-}\leq \text{GHIVIFAWG}\left(H_{1},H_{2},\ldots ,H_{n}\right)\leq H^{+}, \end{array}$$

where *H* ^−^ = {([0,0], [1,1])} and *H* ^+^ = {([1,1], [0,0])}.

**Proof**

The process is omitted here.

### 4.2. HIVIFN Algebraic Aggregation Operators and HIVIFN Einstein Aggregation Operators

Obviously, different *t*-conorms and *t*-norms may lead to different aggregation operators. In the following, HIVIFN algebraic aggregation operators and Einstein aggregation operators are presented based on algebraic norms and Einstein norms.

**Theorem 36**

Let *H*_*j*_ = {⋃_*i*_*j*_=1_^*n*(*H*_*j*_)^〈[*a*_*i*_*j*__, *b*_*i*_*j*__], [*c*_*i*_*j*__, *d*_*i*_*j*__]〉}  (*j* = 1,2,…, *n*) be a collection of HIVIFNs, let *w* = (*w*_1_, *w*_2_,…, *w*_*n*_)^*T*^ be the weight vector of *A*_*j*_  (*j* = 1,2,…, *n*), with *λ* > 0, *w*_*j*_ ≥ 0  (*j* = 1,2,…, *n*), and ∑_*j*=1_^*n*^ *w*_*j*_ = 1, *k*(*x*) = −ln⁡(*x*), and *k* ^−1^(*x*) = *e* ^−*x*^, *l*(*x*) = −ln⁡(1 − *x*), *l* ^−1^(*x*) = 1 − *e* ^−*x*^, *T*(*x*, *y*) = *xy*, and *S*(*x*, *y*) = 1 − ((1 − *x*)(1 − *y*)) be algebraic *t*-conorm and *t*-norm. Then, some HIVIFN algebraic aggregation operators could be obtained as follows.

(1) Hesitant interval-valued intuitionistic fuzzy number algebraic weighted averaging operator is as follows:

(37) HIVIFNAWAw(H1,H2,…,Hn)=⋃i1=1n(H1)⋯⋃in=1n(Hn){〈[1−∏j=1n(1−aij)wj,1−∏j=1n(1−bij)wj],[∏j=1n(cij)wj,∏j=1n(dij)wj]〉}.

(2) Hesitant interval-valued intuitionistic fuzzy number algebraic weighted geometric operator is as follows:

(38) HIVIFNAWGw(H1,H2,…,Hn)=⋃i1=1n(H1)⋯⋃in=1n(Hn){〈[∏j=1n(aij)wj,∏j=1n(bij)wj],[1−∏j=1n(1−cij)wj,1−∏j=1n(1−dij)wj]〉}.

(3) Hesitant interval-valued intuitionistic fuzzy number algebraic weighted arithmetic averaging operator is as follows:
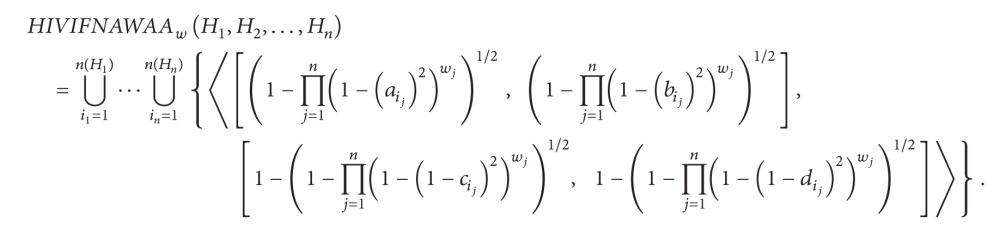
(39)

(4) Hesitant interval-valued intuitionistic fuzzy number algebraic weighted arithmetic geometric operator is as follows:

(40) HIVIFNAWAGw(H1,H2,…,Hn)=⋃i1=1n(H1)⋯⋃in=1n(Hn){〈[1−(1−∏j=1n(1−(1−aij)2)wj)1/2,1−(1−∏j=1n(1−(1−bij)2)wj)1/2],[(1−∏j=1n(1−(cij)2)wj)1/2,(1−∏j=1n(1−(dij)2)wj)1/2]〉}.

(5) Generalized hesitant interval-valued intuitionistic fuzzy number algebraic weighted averaging operator is as follows:
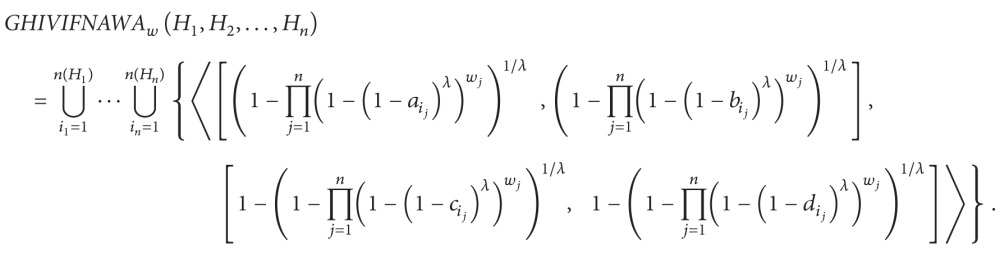
(41)

(6) Generalized hesitant interval-valued intuitionistic fuzzy number algebraic weighted geometric operator is as follows:

(42) GHIVIFNAWGw(H1,H2,…,Hn)=⋃i1=1n(H1)⋯⋃in=1n(Hn){〈[1−(1−∏j=1n(1−(1−aij)λ)wj)1/λ,1−(1−∏j=1n(1−(1−bij)λ)wj)1/λ],[(1−∏j=1n(1−(cij)λ)wj)1/λ,(1−∏j=1n(1−(dij)λ)wj)1/λ]〉}.

In particular, if *λ* = 1, then (41) is reduced to (37) and (42) is reduced to (38); if *λ* = 2, then (41) is reduced to (39) and (42) is reduced to (40).

**Example 37**

Let *H*_1_ = {〈[0.2,0.3], [0.1,0.2]〉, 〈[0.4,0.5], [0.2,0.3]〉} and *H*_2_ = {〈[0.1,0.3], [0.2,0.4]〉} be two HIVIFNs, and let *w* = (0.4,0.6) be the weight of them, and *λ* = 1,2, 5. According to Theorem 36, the following can be calculated:

(1)  HIVIFNAWA_*w*_(*H*_1_, *H*_2_) = {〈[1 − (1−0.2)^0.4^ × (1−0.1)^0.6^, 1 − (1−0.3)^0.4^ × (1−0.3)^0.6^], [0.1^0.4^ × 0.2^0.6^, 0.2^0.4^ × 0.4^0.6^], [1 − (1−0.4)^0.4^ × (1−0.1)^0.6^, 1 − (1−0.5)^0.4^ × (1−0.3)^0.6^], [0.2^0.4^ × 0.2^0.6^, 0.3^0.4^ × 0.4^0.6^]〉} = {〈[0.1414,0.3000], [0.1516,0.3031]〉, 〈[0.2347,0.3881],[0.2000,0.3565]〉}, Consider

(43) HIVIFNAWGw(H1,H2) ={〈[0.1320,0.3000],[0.1614,0.3268]〉, 〈[0.1741,0.3680],[0.2000,0.3618]〉}.

According to Definitions 24, 6, and 8, Consider the following:

(44) S~(HIVIFNAWAw(H1,H2))  ={〈[0.1881,0.3441],[0.1758,0.3298]〉},S~(HIVIFNAWGw(H1,H2))  ={〈[0.1531,0.3340],[0.1807,0.3443]〉}.

$L(\tilde{S}(\text{HIVIFNAW}{\text{A}}_{w}(H_{1},H_{2})))=0.0866,L(\tilde{S}(\text{HIVIFNAW}{\text{G}}_{w}(H_{1},H_{2})))=0.0524$. Thus,

(45) $$\begin{array}{c} \text{HIVIFNAW}{\text{A}}_{w}\left(H_{1},H_{2}\right)>\text{HIVIFNAW}{\text{G}}_{w}\left(H_{1},H_{2}\right). \end{array}$$

(2)  HIVIFNAWAA_*w*_(*H*_1_, *H*_2_) = {〈[0.1487,0.3000], [0.1508,0.2989]〉, 〈[0.2701,0.3972],[0.2000,0.3554]〉};

(46) HIVIFNAWAGw(H1,H2) ={〈[0.1313,0.3000],[0.1677,0.3375]〉, 〈[0.1686,0.3637],[0.2000,0.3642]〉}.

According to Definitions 22, 5, and 6, Consider the following:

(47) S~(HIVIFNAWAAw(H1,H2))  ={〈[0.2094,0.3486],[0.1754,0.3272]〉},S~(HIVIFNAWAGw(H1,H2))  ={〈[0.1500,0.3319],[0.1839,0.3509]〉}.

Consider $L(\tilde{S}(\text{HIVIFNAWA}{\text{A}}_{w}(H_{1},H_{2})))=0.1031,L(\tilde{S}(\text{HIVIFNAWA}{\text{G}}_{w}(H_{1},H_{2})))=0.0456$. Thus,

(48) $$\begin{array}{c} \text{HIVIFNAWA}{\text{A}}_{w}\left(H_{1},H_{2}\right)>\text{HIVIFNAWA}{\text{G}}_{w}\left(H_{1},H_{2}\right). \end{array}$$

(3)  GHIVIFNAWA_*w*_(*H*_1_, *H*_2_) = {〈[0.1680,0.3000], [0.1481,0.2847]〉, 〈[0.3333,0.4262],[0.2000,0.3512]〉};

(49) GHIVIFNAWGw(H1,H2) ={〈[0.1292,0.3000],[0.1813,0.3628]〉, 〈[0.1540,0.3502],[0.2000,0.3720]〉}.

According to Definitions 22, 5, and 6,

(50) S~(GHIVIFNAWAw(H1,H2))  ={〈[0.2507,0.3631],[0.1741,0.3180]〉},S~(GHIVIFNAWGw(H1,H2))  ={〈[0.1416,0.3251],[0.1907,0.3674]〉}.

Consider $L(\tilde{S}(\text{GHIVIFNAW}{\text{A}}_{w}(H_{1},H_{2})))=0.1404,L(\tilde{S}(\text{GHIVIFNAW}{\text{G}}_{w}(H_{1},H_{2})))=0.0459$. Thus,

(51) $$\begin{array}{c} \text{GHIVIFNAW}{\text{A}}_{w}\left(H_{1},H_{2}\right)>\text{GHIVIFNAW}{\text{G}}_{w}\left(H_{1},H_{2}\right). \end{array}$$

In the three cases listed above, the aggregation results by using the GHIVIFNAWA operator are greater than the aggregation results by utilizing the GHIVIFNAWG operator.

**Theorem 38**

Let *H*_*j*_ = {⋃_*i*_*j*_=1_^*n*(*H*_*j*_)^〈[*a*_*i*_*j*__, *b*_*i*_*j*__], [*c*_*i*_*j*__, *d*_*i*_*j*__]〉}  (*j* = 1,2,…, *n*) be a collection of HIVIFNs, and let *w* = (*w*_1_, *w*_2_,…, *w*_*n*_)^*T*^ be the weight vector of *A*_*j*_  (*j* = 1,2,…, *n*), with *λ* > 0, *w*_*j*_ ≥ 0  (*j* = 1,2,…, *n*), and ∑_*j*=1_^*n*^ *w*_*j*_ = 1, *k*(*x*) = ln⁡((2 − *x*)/*x*), and *k* ^−1^(*x*) = 2/(*e* ^*x*^ + 1), *l*(*x*) = ln⁡((2 − (1 − *x*))/(1 − *x*)), *l* ^−1^(*x*) = 1 − (2/(*e* ^*x*^ + 1)),*T*(*x*, *y*) = *xy*/(1 + (1 − *x*)(1 − *y*)), and *S*(*x*, *y*) = (*x* + *y*)/(1 + *xy*) be the Einstein *t*-conorm and *t*-norm, respectively. Then, some HIVIFN Einstein aggregation operators could be obtained as follows.

(1) Hesitant interval-valued intuitionistic fuzzy number Einstein weighted averaging operator is as follows:

(52) HIVIFNEWAw(H1,H2,…,Hn)=⋃i1=1n(H1)⋯⋃in=1n(Hn){[∏j=1n((1+aij)/(1−aij))wj−1∏j=1n((1+aij)/(1−aij))wj+1,∏j=1n((1+bij)/(1−bij))wj−1∏j=1n((1+bij)/(1−bij))wj+1],[2∏j=1n((2−cij)/cij)wj+1,2∏j=1n((2−dij)/dij)wj+1]}.

(2) Hesitant interval-valued intuitionistic fuzzy number Einstein weighted geometric operator is as follows:

(53) HIVIFNEWGw(H1,H2,…,Hn)=⋃i1=1n(H1)⋯⋃in=1n(Hn){[2∏j=1n((2−aij)/aij)wj+1,2∏j=1n((2−bij)/bij)wj+1],[∏j=1n((1+cij)/(1−cij))wj−1∏j=1n((1+cij)/(1−cij))wj+1,∏j=1n((1+dij)/(1−dij))wj−1∏j=1n((1+dij)/(1−dij))wj+1]}.

(3) Hesitant interval-valued intuitionistic fuzzy number Einstein weighted arithmetic averaging operator is as follows:
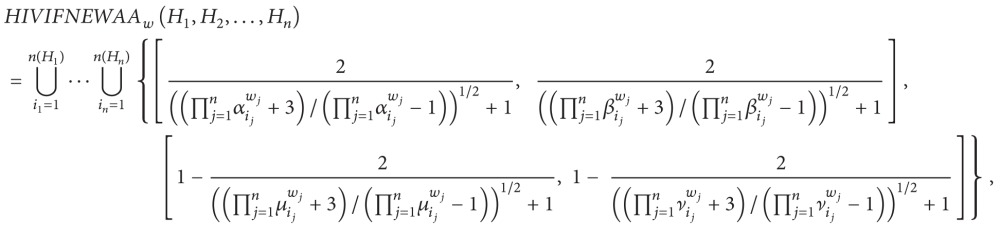
(54)
where

(55) $$\begin{array}{c} \alpha_{i_{j}}=\left(\frac{a_{i_{j}}^{2}-a_{i_{j}}+1}{1-a_{i_{j}}}\right),\;\;\beta_{i_{j}}=\left(\frac{b_{i_{j}}^{2}-b_{i_{j}}+1}{1-b_{i_{j}}}\right),\\ \mu_{i_{j}}=\left(\frac{c_{i_{j}}^{2}-c_{i_{j}}+1}{c_{i_{j}}}\right),\;\;\nu_{i_{j}}=\left(\frac{d_{i_{j}}^{2}-d_{i_{j}}+1}{d_{i_{j}}}\right). \end{array}$$

(4) Hesitant interval-valued intuitionistic fuzzy number Einstein weighted arithmetic geometric operator is as follows:
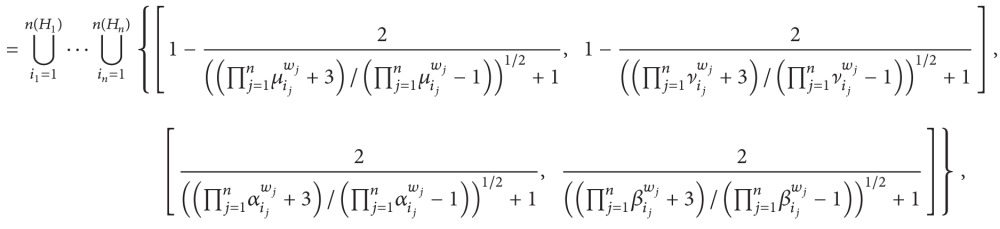
(56)
where

(57) $$\begin{array}{c} \mu_{i_{j}}=\left(\frac{a_{i_{j}}^{2}-a_{i_{j}}+1}{a_{i_{j}}}\right),\;\;\nu_{i_{j}}=\left(\frac{b_{i_{j}}^{2}-b_{i_{j}}+1}{b_{i_{j}}}\right),\\ \alpha_{i_{j}}=\left(\frac{c_{i_{j}}^{2}-c_{i_{j}}+1}{1-c_{i_{j}}}\right),\;\;\beta_{i_{j}}=\left(\frac{d_{i_{j}}^{2}-d_{i_{j}}+1}{1-d_{i_{j}}}\right). \end{array}$$

(5) Generalized hesitant interval-valued intuitionistic fuzzy number Einstein weighted averaging operator is as follows:
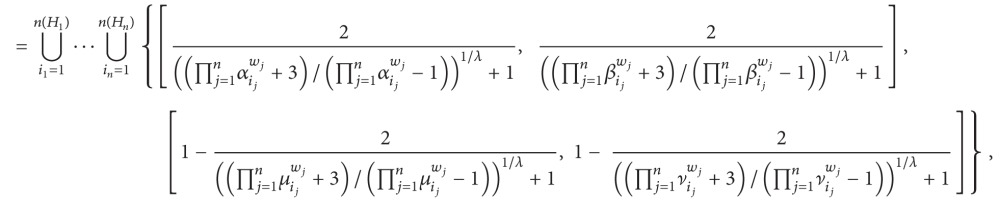
(58)where

(59) $$\begin{array}{c} \alpha_{i_{j}}=\left(\frac{1+\left(2/\left({\left(\left(2-a_{i_{j}}\right)/a_{i_{j}}\right)}^{\lambda }+1\right)\right)}{1-\left(2/\left({\left(\left(2-a_{i_{j}}\right)/a_{i_{j}}\right)}^{\lambda }+1\right)\right)}\right),\\ \beta_{i_{j}}=\left(\frac{1+\left(2/\left({\left(\left(2-b_{i_{j}}\right)/b_{i_{j}}\right)}^{\lambda }+1\right)\right)}{1-\left(2/\left({\left(\left(2-b_{i_{j}}\right)/b_{i_{j}}\right)}^{\lambda }+1\right)\right)}\right),\\ \mu_{i_{j}}=\left(\frac{1+\left(2/\left({\left(\left(1+c_{i_{j}}\right)/\left(1-c_{i_{j}}\right)\right)}^{\lambda }+1\right)\right)}{1-\left(2/\left({\left(\left(1+c_{i_{j}}\right)/\left(1-c_{i_{j}}\right)\right)}^{\lambda }+1\right)\right)}\right),\\ \nu_{i_{j}}=\left(\frac{1+\left(2/\left({\left(\left(1+d_{i_{j}}\right)/\left(1-d_{i_{j}}\right)\right)}^{\lambda }+1\right)\right)}{1-\left(2/\left({\left(\left(1+d_{i_{j}}\right)/\left(1-d_{i_{j}}\right)\right)}^{\lambda }+1\right)\right)}\right). \end{array}$$

(6) Generalized hesitant interval-valued intuitionistic fuzzy number Einstein weighted geometric operator is as follows:
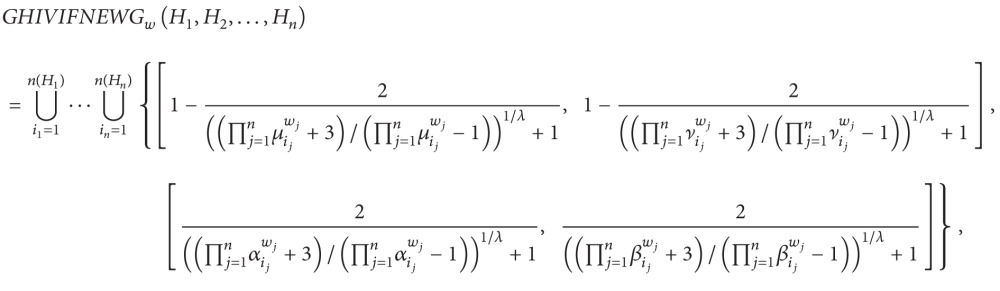
(60)where

(61) $$\begin{array}{c} \mu_{i_{j}}=\left(\frac{1+\left(2/\left({\left(\left(1+a_{i_{j}}\right)/\left(1-a_{i_{j}}\right)\right)}^{\lambda }+1\right)\right)}{1-\left(2/\left({\left(\left(1+a_{i_{j}}\right)/\left(1-a_{i_{j}}\right)\right)}^{\lambda }+1\right)\right)}\right),\\ \nu_{i_{j}}=\left(\frac{1+\left(2/\left({\left(\left(1+b_{i_{j}}\right)/\left(1-b_{i_{j}}\right)\right)}^{\lambda }+1\right)\right)}{1-\left(2/\left({\left(\left(1+b_{i_{j}}\right)/\left(1-b_{i_{j}}\right)\right)}^{\lambda }+1\right)\right)}\right),\\ \alpha_{i_{j}}=\left(\frac{1+\left(2/\left({\left(\left(2-c_{i_{j}}\right)/c_{i_{j}}\right)}^{\lambda }+1\right)\right)}{1-\left(2/\left({\left(\left(2-c_{i_{j}}\right)/c_{i_{j}}\right)}^{\lambda }+1\right)\right)}\right),\\ \beta_{i_{j}}=\left(\frac{1+\left(2/\left({\left(\left(2-d_{i_{j}}\right)/d_{i_{j}}\right)}^{\lambda }+1\right)\right)}{1-\left(2/\left({\left(\left(2-d_{i_{j}}\right)/d_{i_{j}}\right)}^{\lambda }+1\right)\right)}\right). \end{array}$$

In particular, if *λ* = 1, then (58) is reduced to (52) and (60) is reduced to (53); if *λ* = 2, then (58) is reduced to (54) and (60) is reduced to (56).

### 4.3. The MCDM Approach Based on the GHIVIFNWA and GHIVIFNWG Operators

Let *A* = {*a*_1_, *a*_2_,…,*a*_*m*_} be a finite set of alternatives, and let *C* = {*c*_1_, *c*_2_,…,*c*_*n*_} be a finite set of criteria, whose criteria weight vector is *w* = (*w*_1_, *w*_2_,…, *w*_*n*_), where *w*_*j*_ ≥ 0 (*j* = 1,2,…, *n*), ∑_*j*=1_^*n*^ *w*_*j*_ = 1. Let $R={\left({\tilde{\alpha }}_{ij}\right)}_{m\times n}$ be the hesitant interval-valued intuitionistic fuzzy decision matrix, where ${\tilde{\alpha }}_{ij}$ is a criterion value, denoted by HIVIFNs. The characteristics of the alternatives *a*_*i*_ (*i* = 1,2,…, *m*) with respect to the attributes *c*_*j*_ (*j* = 1,2,…, *n*) can be denoted by ${\tilde{\alpha }}_{ij}=\left\{{⋃}_{r=1}^{n({\tilde{\alpha }}_{ij})}\langle \left[a_{{\tilde{\alpha }}_{ij}}^{r},b_{{\tilde{\alpha }}_{ij}}^{r}\right],\left[c_{{\tilde{\alpha }}_{ij}}^{r},d_{{\tilde{\alpha }}_{ij}}^{r}\right]\rangle \right\}$. In the following, we propose one approach to rank and select the most desirable alternative(s). The procedure of this approach is shown as follows.

**Step 1**

Aggregate the HIVIFNs ${\tilde{\alpha }}_{ij}\;(i=1,2,\ldots ,n,j=1,2,\ldots ,n)$ of the alternative *a*_*i*_ (*i* = 1,2,…, *m*).

Utilize the GHIVIFNWA or GHIVIFNWG operator to obtain the overall values *y*_*i*_ for the alternatives *a*_*i*_ (*i* = 1,2,…, *m*), respectively; that is,

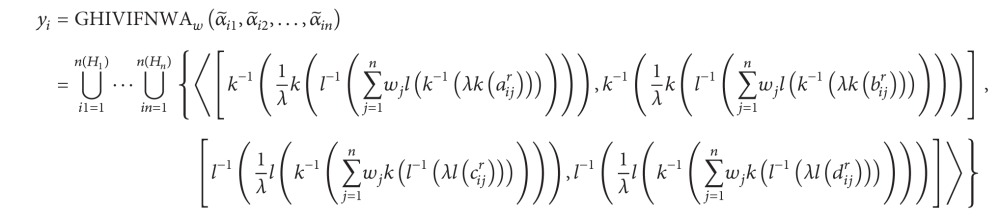
(62)
or

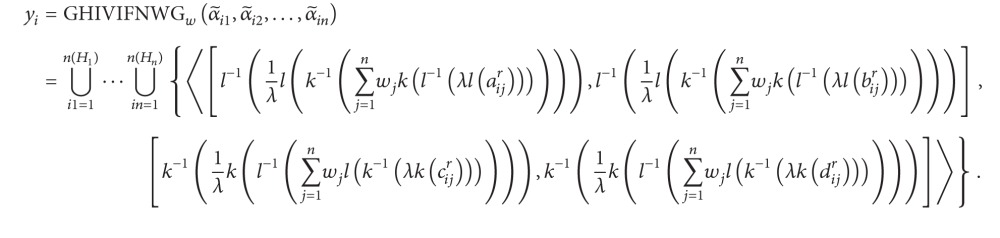
(63)

**Step 2**

Calculate the score values. According to Definition 22, the score of overall values $\tilde{S}(y_{i})$ (*i* = 1,2,…, *m*) could be calculated.

**Step 3**

Rank the preference order of all alternatives *a*_*i*_ (*i* = 1,2,…, *m*). $L\left(\tilde{S}\left(y_{i}\right)\right)\;(i=1,2,\ldots ,m)$ could be obtained according to Definition 5. The greater the value of $L(\tilde{S}(y_{i}))$ is, the better the alternative *a*_*i*_  (*i* = 1,2,…, *m*) will be.

**Step 4**

Select the optimal one(s).

## 5. Illustrative Example

In this section, the proposed approach and one existing method are utilized to evaluate four companies with hesitant interval-valued intuitionistic fuzzy information.

The enterprise's board of directors intends to find an automobile company and establish a foundation for deeper and more extensive cooperation with it in the following five years. Suppose there are four possible projects *a*_*i*_ (*i* = 1,2, 3,4) to be evaluated. It is necessary to compare these companies and rank them in terms of their importance. Four criteria, suggested by the Balanced Scorecard methodology, could be taken into account (it should be noted that all of them are of the maximization type): *c*_1_: economy, *c*_2_: comfort, *c*_3_: design, and *c*_4_: safety. And suppose that the weight vector of the criteria is *w* = (0.2,0.3,0.15,0.35). The decision-makers are required to provide their evaluation of the company *a*_*i*_ under the criterion *c*_*j*_ (*i* = 1,2, 3,4, *j* = 1,2, 3,4). The hesitant interval-valued intuitionistic fuzzy decision matrix $R={\left({\tilde{\alpha }}_{ij}\right)}_{4\times 4}$ is shown in Table 1, where ${\tilde{\alpha }}_{ij}$ (*i* = 1,2, 3,4, *j* = 1,2, 3,4) are in the form of HIVIFNs.

### 5.1. Illustration of the Proposed Approach

In order to get the optimal alternative(s), the following steps are involved.

**Step 1**

Aggregate the HIVIFNs ${\tilde{\alpha }}_{ij}\;(i=1,2,\ldots ,n,j=1,2,\ldots ,n)$ of the alternative *a*_*i*_  (*i* = 1,2,…, *m*).

For the convenience of analysis and computation, we use hesitant interval-valued intuitionistic fuzzy algebraic aggregation operators to fuse the attribute values which are represented in the form of HIVIFNs in MCDM problems. Let *k*(*x*) = −ln⁡(*x*), and let *k* ^−1^(*x*) = *e* ^−*x*^, *l*(*x*) = −ln⁡(1 − *x*), *l* ^−1^(*x*) = 1 − *e* ^−*x*^, and *T*(*x*, *y*) = *xy*, and *S*(*x*, *y*) = 1 − ((1 − *x*)(1 − *y*)) be algebraic *t*-conorm and *t*-norm. Then, the GHIVIFNWA or GHIVIFNWG operators are, respectively, reduced to the GHIVIFNAWA or GHIVIFNAWG operators, and (62) and (63) are reduced to the following expression; that is,(64)
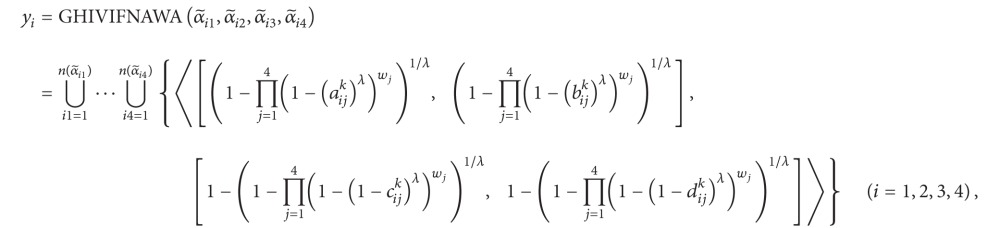

or
(65)
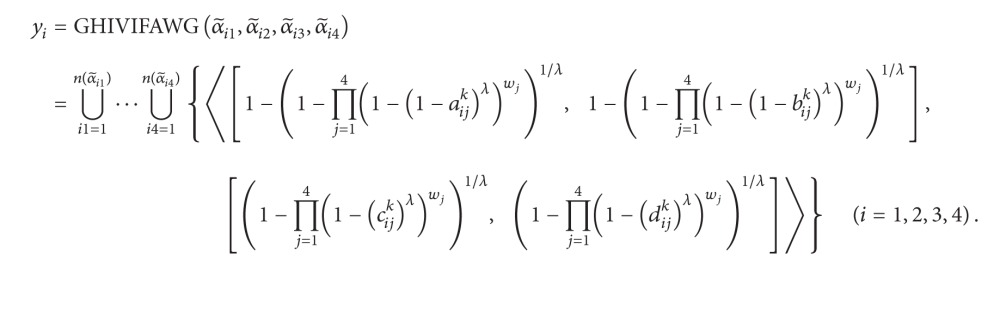

Let *λ* = 2, and according to the formula listed above, the overall HIVIFNs *y*_*i*_ of the alternatives *a*_*i*_  (*i* = 1,2, 3,4) could be obtained and shown in Table 2.

**Step 2**

Based on Definition 22 and Table 2, the score values of overall HIVIFNs $\tilde{S}(y_{i})$ (*i* = 1,2, 3,4) can be obtained and shown in Table 3.

**Step 3**

Rank all the alternatives *a*_*i*_  (*i* = 1,2, 3,4) in accordance with the scores $\tilde{S}\left(y_{i}\right)\;(i=1,2,3,4)$ of the aggregated hesitant interval-valued intuitionistic fuzzy values by using Definitions 5 and 6. From Table 3, the following results can be obtained.

**Case 1**

The GHIVIFAWA operator is as follows:

(66) $$\begin{array}{c} L\left(\tilde{S}\left(y_{1}\right)\right)=0.3725,\;\;L\left(\tilde{S}\left(y_{2}\right)\right)=0.5612,\\ L\left(\tilde{S}\left(y_{3}\right)\right)=0.5032,\;\;L\left(\tilde{S}\left(y_{4}\right)\right)=0.5681. \end{array}$$

So the final ranking of alternatives is *a*_4_≻*a*_2_≻*a*_3_≻*a*_1_.

**Case 2**

The GHIVIFAWG operator is as follows:

(67) $$\begin{array}{c} L\left(\tilde{S}\left(y_{1}\right)\right)=0.3049,\;\;L\left(\tilde{S}\left(y_{2}\right)\right)=0.4990,\\ L\left(\tilde{S}\left(y_{3}\right)\right)=0.4300;\;\;L\left(\tilde{S}\left(y_{4}\right)\right)=0.4669. \end{array}$$

So the final ranking of alternatives is *a*_2_≻*a*_4_≻*a*_3_≻*a*_1_.

**Step 4**

Select the best one(s). In Step 3, if the GHIVIFNAWA operator is utilized, then the optimal alternative is *a*_2_ while the worst alternative is *a*_1_; if the GHIVIFNAWG operator is used, then the optimal alternative is *a*_4_ while the worst alternative is *a*_1_.

### 5.2. Sensitivity Analysis

In Step 1, two aggregation operators can be used and the sensitivity analysis will be conducted in these following cases.

(1) The hesitant interval-valued intuitionistic fuzzy algebraic aggregation operators in Step 1  are illustrated as follows.

In order to investigate the influence of *λ* on the ranking of alternatives, different *λ* are utilized. The ranking results are shown in Tables 4 and 5.

From Tables 4 and 5, the GHIVIFNAWA and GHIVIFNAWG operators have produced different rankings of the alternatives. However, for each operator, the rankings obtained are consistent as *λ* changes. Moreover, *a*_4_ or *a*_2_ is always the optimal one while the worst one is always *a*_1_.

(2) The hesitant interval-valued intuitionistic fuzzy Einstein aggregation operators in Step 1  are illustrated as follows.

Let *k*(*x*) = ln⁡((2 − *x*)/*x*), and let *k* ^−1^(*x*) = 2/(*e* ^*x*^ + 1),  *l*(*x*) = ln⁡((2 − (1 − *x*))/(1 − *x*)), *l* ^−1^(*x*) = 1 − (2/(*e* ^*x*^ + 1)), *T*(*x*, *y*) = *xy*/(1 + (1 − *x*)(1 − *y*)), and *S*(*x*, *y*) = (*x* + *y*)/(1 + *xy*) be the Einstein *t*-conorm and *t*-norm, respectively. Then, the GHIVIFNWA and GHIVIFNWG operators are, respectively, reduced to the GHIVIFNEWA and GHIVIFNEWG operators. According to (58) and (60), the following results could be obtained and shown in Tables 6 and 7.

From Tables 6 and 7, the GHIVIFNEWA and GHIVIFNEWG operators have produced different rankings of the alternatives. Furthermore, for each operator, the aggregation parameter *λ* also leads to different aggregation results, but the final rankings of alternatives are the same as the parameter changes. What is more, regardless of using the GHIVIFNEWA and GHIVIFNEWG operators, is that *a*_4_ or *a*_2_ is always the optimal one while the worst one is always *a*_1_.

It can be concluded from the sensitivity analysis that different *t*-conorms and *t*-norms could lead to different aggregation results. However, the rankings using each operator are consistent.

### 5.3. Comparison Analysis

Based on the same decision-making problem, if the method of Chen et al. [16] is employed, HIVIFNs are transformed to IVIFNs by using the score function firstly, and then IVIFNs could be aggregated by the interval-valued intuitionistic fuzzy weighted aggregation operators, proposed by Chen et al. [16].

Based on Definition 3 and ∑_*i*=1_^4^ *w*_*i*_ = 1, the interval-valued intuitionistic fuzzy weighted average values of all alternatives could be obtained as follows:

(68) IVIFWAw(a11,a12,a13,a14)=∑i=14[[a1i,b1i],[1−d1i,1−c1i]]×wi∑i=1nwi=[[∑i=14a1iwi,∑i=14b1iwi],[∑i=1n(1−d1i)wi,∑i=1n(1−c1i)wi]]=[[0.4×0.2+0.3+0.72×0.3+0.5×0.15+0.4×0.35,0.5×0.2+0.4+0.72×0.3+0.6×0.15+0.4×0.35],[(1−0.3)×0.2+(1−0.2+0.32)×0.3+(1−0.4)×0.15+(1−0.2)×0.35,(1−0.2)×0.2+(1−0.1+0.32)×0.3+(1−0.3)×0.15+(1−0.2)×0.35]]=[[0.445,0.495],[0.735,0.785]]=〈[0.445,0.495],[1−0.785,1−0.735]〉=〈[0.445,0.495],[0.215,0.265]〉,IVIFWAw(a21,a22,a23,a24)  =[[0.560,0.660],[0.635,0.87]]  =〈[0.560,0.660],[0.13,0.365]〉,IVIFWAw(a31,a32,a33,a34) =[[0.515,0.615],[0.695,0.830]] =〈[0.515,0.615],[0.170,0.305]〉,IVIFWAw(a41,a42,a43,a44)  =[[0.578,0.648],[0.743,0.813]]  =〈[0.578,0.648],[0.187,0.257]〉.

According to Definitions 5 and 6,

(69) $$\begin{array}{c} L\left(\text{IVIFW}{\text{A}}_{w}\left(a_{11},a_{12},a_{13},a_{14}\right)\right)=0.343,\\ L\left(\text{IVIFW}{\text{A}}_{w}\left(a_{21},a_{22},a_{23},a_{24}\right)\right)=0.519,\\ L\left(\text{IVIFW}{\text{A}}_{w}\left(a_{31},a_{32},a_{33},a_{34}\right)\right)=0.486,\\ L\left(\text{IVIFW}{\text{A}}_{w}\left(a_{41},a_{42},a_{43},a_{44}\right)\right)=0.528. \end{array}$$

So *a*_4_≻*a*_2_≻*a*_3_≻*a*_1_ and the best optimal one is *a*_4_. The ranking here is the same as the result using the GHIVIFNAWA and GHIVIFNAWA operators.

According to the calculation results, although the existing method can produce the same result as the proposed method, the method being compared has a problem that how to transform HIVIFNs to IVIFNs in the first step could avoid information loss in the process of transformation. By contrast, the proposed approach based on different *t*-conorms and *t*-norms can be used to deal with different relationships among the aggregated arguments, could handle MCDM problems in a flexible and objective manner under hesitant interval-valued intuitionistic fuzzy environment, and can provide more choices for decision-makers. Additionally, different *t*-conorms and *t*-norms and aggregation operators could be chosen in the practical decision-making process. At the same time, different results may be produced, which reflected the preferences of decision-makers. Therefore, the developed approach can produce better results than the existing method.

## 6. Conclusion

HFSs are the extension of traditional fuzzy sets, and their membership degree of an element is a set of several possible values between 0 and 1. IVIFSs can describe the fuzzy concept “neither this nor that,” and the membership degrees and nonmembership degrees of IVIFSs are not only real numbers but interval values, respectively. Precise numerical values in HFSs can be replaced by IVIFSs, which provide more preference information for decision-makers. In this paper, the definition of HIVIFSs was developed and applied to the MCDM problems, in which the evaluation values of alternatives on criteria were expressed with HIVIFNs. Furthermore, based on *t*-conorms and *t*-norms, some aggregation operators, namely, the HIVIFNWA and HIVIFNWG, HIVIFNWAA and HIVIFNWGA, and GHIVIFNWA and GHIVIFNWG operators, were proposed, respectively. Their properties were discussed in detail as well. In particular, the corresponding hesitant interval-valued intuitionistic fuzzy algebraic aggregation operators based on algebraic *t*-conorm and *t*-norm and hesitant interval-valued intuitionistic fuzzy Einstein aggregation operators based on Einstein *t*-conorm and *t*-norm were presented. In addition, different aggregation operators were utilized to fuse the hesitant interval-valued intuitionistic fuzzy information to get the overall HIVIFNs of alternatives and the ranking of all given alternatives. At last, the example was presented to illustrate the fuzzy decision-making process, and the sensitivity analysis and comparison analysis were conducted to enrich the paper. The prominent feature of the proposed method is that it could provide a useful and flexible way to efficiently facilitate decision-makers under a hesitant interval-valued intuitionistic fuzzy environment, and the related calculations are simple. Hence, it has enriched and developed the theories and methods of MCDM problems and also has provided a new idea for solving MCDM problems. In the future research, the distance and similarity measure of HIVIFSs will be studied to solve MCDM problems.

## Figures and Tables

**Table 1:** Hesitant interval-valued intuitionistic fuzzy decision matrix $R={\left({\tilde{\alpha }}_{ij}\right)}_{4\times 4}$.

	*c*_1_	*c*_2_	*c*_3_	*c*_4_
*a*_1_	{〈[0.4,0.5], [0.2,0.3]〉}	{〈[0.1,0.2], [0.3,0.4]〉, 〈0.7,0.3〉}	{〈[0.5,0.6], [0.3,0.4]〉}	{〈0.4,0.2〉}
*a*_2_	{〈[0.7,0.8], [0.1,0.2]〉}	{〈[0.3,0.4], [0.2,0.3]〉, 〈[0.6,0.7], [0.1,0.3]〉}	{〈[0.5,0.6], [0.2,0.4]〉}	{〈[0.6,0.7], [0.1,0.3]〉}
*a*_3_	{〈[0.4,0.5], [0.3,0.4]〉}	{〈[0.4,0.5], [0.2,0.3]〉}	{〈[0.7,0.8], [0.1,0.2]〉}	{〈[0.6,0.7], [0.1,0.3]〉}
*a*_4_	{〈[0.5,0.6], [0.2,0.3]〉}	{〈0.6,0.3〉}	{〈[0.2,0.3], [0.1,0.2]〉, 〈[0.5,0.6], [0.2,0.3]〉}	{〈[0.7,0.8], [0.1,0.2]〉}

**Table 2:** The overall HIVIFNs of alternatives.

*λ* = 2	GHIVIFAWA (HIVIFAWA)	GHIVIFNWG (HIVIFAWG)
*y*_1_	{〈[0.3925,0.4601], [0.1712,0.2389]〉, 〈[0.5376,0.5668], [0.2391,0.2700]〉}	{〈[0.3373,0.4415], [0.1971,0.2628]〉, 〈[0.4788,0.5150], [0.2507,0.2896]〉}
*y*_2_	{〈[0.5513,0.6545], [0.1359,0.2874]〉, 〈[0.6113,0.7126], [0.1107,0.2874]〉}	{〈[0.4732,0.5731], [0.1537,0.3020]〉, 〈[0.5990,0.6980], [0.1207,0.3020]〉}
*y*_3_	{〈[0.5398,0.6427], [0.1517,0.2975]〉}	{〈[0.4921,0.5911], [0.1884,0.3118]〉}
*y*_4_	{〈[0.5929,0.6696], [0.1576,0.2440]〉, 〈[0.6125,0.6905], [0.1751,0.2594]〉}	{〈[0.5880,0.6539], [0.2123,0.2698]〉, 〈[0.4938,0.5348], [0.2016,0.2556]〉}

**Table 3:** The score values of overall HIVIFNs.

GHIVIFAWA	*λ* = 2 (HIVIFAWA)	GHIVIFAWG	*λ* = 2 (HIVIFAWG)
$\tilde{S}(y_{1})$	{〈[0.4651,0.5135], [0.2052,0.2545]〉}	$\tilde{S}(y_{1})$	{〈[0.4081,0.4783], [0.2239,0.2762]〉}
$\tilde{S}(y_{2})$	{〈[0.5813,0.6836], [0.1233,0.2874]〉}	$\tilde{S}(y_{2})$	{〈[0.5361,0.6356], [0.1372,0.3020]〉}
$\tilde{S}(y_{3})$	{〈[0.5398,0.6427], [0.1517,0.2975]〉}	$\tilde{S}(y_{3})$	{〈[0.4921,0.5911], [0.1884,0.3118]〉}
$\tilde{S}(y_{4})$	{〈[0.6027,0.6801], [0.1664,0.2517]〉}	$\tilde{S}(y_{4})$	{〈[0.5409,0.5944], [0.2070,0.2627]〉}

**Table 4:** Rankings obtained using the GHIVIFNAWA operator.

*λ*	*a*_1_	*a*_2_	*a*_3_	*a*_4_	Rankings
*λ* = 1	0.3627	0.5529	0.4927	0.5581	*a*_4_≻*a*_2_≻*a*_3_≻*a*_1_
*λ* = 2	0.3725	0.5612	0.5032	0.5681	*a*_4_≻*a*_2_≻*a*_3_≻*a*_1_
*λ* = 5	0.3914	0.5836	0.5363	0.5940	*a*_4_≻*a*_2_≻*a*_3_≻*a*_1_
*λ* = 10	0.4493	0.6105	0.5804	0.6263	*a*_4_≻*a*_2_≻*a*_3_≻*a*_1_
*λ* = 20	0.4949	0.6443	0.6287	0.6624	*a*_4_≻*a*_2_≻*a*_3_≻*a*_1_
*λ* = 30	0.5152	0.6642	0.6537	0.6788	*a*_4_≻*a*_2_≻*a*_3_≻*a*_1_

**Table 5:** Rankings obtained using the GHIVIFNAWG operator.

*λ*	*a*_1_	*a*_2_	*a*_3_	*a*_4_	Rankings
*λ* = 1	0.3291	0.5148	0.4329	0.4482	*a*_2_≻*a*_4_≻*a*_3_≻*a*_1_
*λ* = 2	0.3049	0.4990	0.4300	0.4669	*a*_2_≻*a*_4_≻*a*_3_≻*a*_1_
*λ* = 5	0.2819	0.4517	0.3804	0.4076	*a*_2_≻*a*_4_≻*a*_3_≻*a*_1_
*λ* = 10	0.2428	0.3995	0.2877	0.3338	*a*_2_≻*a*_4_≻*a*_3_≻*a*_1_
*λ* = 20	0.2071	0.3523	0.2824	0.2993	*a*_2_≻*a*_4_≻*a*_3_≻*a*_1_
*λ* = 30	0.1858	0.3372	0.2608	0.2699	*a*_2_≻*a*_4_≻*a*_3_≻*a*_1_

**Table 6:** Rankings obtained using the GHIVIFNEWA operator.

*λ*	*a*_1_	*a*_2_	*a*_3_	*a*_4_	Rankings
*λ* = 1	0.4143	0.5478	0.4864	0.5515	*a*_4_≻*a*_2_≻*a*_3_≻*a*_1_
*λ* = 2	0.3708	0.5586	0.4994	0.5641	*a*_4_≻*a*_2_≻*a*_3_≻*a*_1_
*λ* = 5	0.2752	0.5955	0.5559	0.6075	*a*_4_≻*a*_2_≻*a*_3_≻*a*_1_
*λ* = 10	0.4751	0.6317	0.6113	0.6505	*a*_4_≻*a*_2_≻*a*_3_≻*a*_1_
*λ* = 20	0.5146	0.6669	0.6571	0.6812	*a*_4_≻*a*_2_≻*a*_3_≻*a*_1_
*λ* = 30	0.5295	0.6825	0.6759	0.6925	*a*_4_≻*a*_2_≻*a*_3_≻*a*_1_

**Table 7:** Rankings obtained using the GHIVIFNEWG operator.

*λ*	*a*_1_	*a*_2_	*a*_3_	*a*_4_	Rankings
*λ* = 1	0.3342	0.5213	0.4551	0.5127	*a*_2_≻*a*_4_≻*a*_3_≻*a*_1_
*λ* = 2	0.3156	0.4967	0.4093	0.4274	*a*_2_≻*a*_4_≻*a*_3_≻*a*_1_
*λ* = 5	0.2669	0.4315	0.3622	0.3760	*a*_2_≻*a*_4_≻*a*_3_≻*a*_1_
*λ* = 10	0.2262	0.3767	0.3048	0.3184	*a*_2_≻*a*_4_≻*a*_3_≻*a*_1_
*λ* = 20	0.1951	0.3414	0.2905	0.2986	*a*_2_≻*a*_4_≻*a*_3_≻*a*_1_
*λ* = 30	0.1745	0.3034	0.2578	0.2602	*a*_2_≻*a*_4_≻*a*_3_≻*a*_1_

## References

[1] Zadeh LA (1965). Fuzzy sets. *Information and Control*.

[2] Atanassov KT Intuitionistic fuzzy sets.

[3] Atanassov KT (1986). Intuitionistic fuzzy sets. *Fuzzy Sets and Systems*.

[4] Atanassov K, Gargov G (1989). Interval valued intuitionistic fuzzy sets. *Fuzzy Sets and Systems*.

[5] Xu ZS (2007). Intuitionistic fuzzy aggregation operators. *IEEE Transactions on Fuzzy Systems*.

[6] Xu ZS, Chen J (2007). An approach to group decision making based on interval-valued intuitionistic judgment matrices. *System Engineering Theory and Practice*.

[7] Ye J (2009). Multicriteria fuzzy decision-making method based on a novel accuracy function under interval-valued intuitionistic fuzzy environment. *Expert Systems with Applications*.

[8] Wu J, Huang HB, Cao QW (2013). Research on AHP with interval-valued intuitionistic fuzzy sets and its application in multi-criteria decision making problems. *Applied Mathematical Modelling*.

[9] Atanassov KT (1994). Operators over interval valued intuitionistic fuzzy sets. *Fuzzy Sets and Systems*.

[10] Lee W A novel method for ranking interval-valued intuitionistic fuzzy numbers and its application to decision making.

[11] Lee W An enhanced multicriteria decision-making method of machine design schemes under interval-valued intuitionistic fuzzy environment.

[12] Li DF (2010). TOPSIS-based nonlinear-programming methodology for multiattribute decision making with interval-valued intuitionistic fuzzy sets. *IEEE Transactions on Fuzzy Systems*.

[13] Park JH, Park IY, Kwun YC, Tan XG (2011). Extension of the TOPSIS method for decision making problems under interval-valued intuitionistic fuzzy environment. *Applied Mathematical Modelling*.

[14] Chen TY, Wang HP, Lu YY (2011). A multicriteria group decision-making approach based on interval-valued intuitionistic fuzzy sets: a comparative perspective. *Expert Systems with Applications*.

[15] Nayagam VLG, Sivaraman G (2011). Ranking of interval-valued intuitionistic fuzzy sets. *Applied Soft Computing Journal*.

[16] Chen SM, Lee LW, Liu HC, Yang SW (2012). Multiattribute decision making based on interval-valued intuitionistic fuzzy values. *Expert Systems with Applications*.

[17] Meng FY, Tan CQ, Zhang Q (2013). The induced generalized interval-valued intuitionistic fuzzy hybrid Shapley averaging operator and its application in decision making. *Knowledge-Based Systems*.

[18] Torra V, Narukawa Y On hesitant fuzzy sets and decision.

[19] Xia MM, Xu ZS (2011). Hesitant fuzzy information aggregation in decision making. *International Journal of Approximate Reasoning*.

[20] Zhu B, Xu ZS, Xia MM (2012). Hesitant fuzzy geometric Bonferroni means. *Information Sciences*.

[21] Wei GW (2012). Hesitant fuzzy prioritized operators and their application to multiple attribute decision making. *Knowledge-Based Systems*.

[22] Xu ZS, Xia MM (2011). Distance and similarity measures for hesitant fuzzy sets. *Information Sciences*.

[23] Xu ZS, Xia MM (2011). On distance and correlation measures of hesitant fuzzy information. *International Journal of Intelligent Systems*.

[24] Chen N, Xu ZS, Xia MM (2013). Correlation coefficients of hesitant fuzzy sets and their applications to clustering analysis. *Applied Mathematical Modeling*.

[25] Peng DH, Gao CY, Gao ZF (2013). Generalized hesitant fuzzy synergetic weighted distance measures and their application to multiple criteria decision-making. *Applied Mathematical Modeling*.

[26] Zhang N, Wei GW (2013). Extension of VIKOR method for decision making problem based on hesitant fuzzy set. *Applied Mathematical Modeling*.

[27] Zhang ZM (2013). Hesitant fuzzy power aggregation operators and their application to multiple attribute group decision making. *Information Sciences*.

[28] Chen N, Xu ZS, Xia MM (2013). Interval-valued hesitant preference relation relations and their applications to group decision making. *Knowledge-Based Systems*.

[29] Wei GW (2013). Some hesitant interval-valued fuzzy aggregation operators and their applications to multiple attribute decision making. *Knowledge-Based Systems*.

[30] Wei GW, Zhao XF (2013). Induced hesitant interval-valued fuzzy Einstein aggregation operators and their application to multiple attribute decision making. *Journal of Intelligent and Fuzzy Systems*.

[31] Zhu B, Xu ZS, Xia MM (2012). Dual hesitant fuzzy sets. *Journal of Applied Mathematics*.

[32] Sengupta A, Pal TK (2000). On comparing interval numbers. *European Journal of Operational Research*.

[33] Chen PY (2005). An interval estimation for the number of signals. *Signal Processing*.

[34] Xu ZS (2008). Dependent uncertain ordered weighted aggregation operators. *Information Fusion*.

[35] Karnik NN, Mendel JM (2001). Centroid of a type-2 fuzzy set. *Information Sciences*.

[36] Nayagam VLG, Muralikrishnan S, Sivaraman G (2011). Multi-criteria decision-making method based on interval-valued intuitionistic fuzzy sets. *Expert Systems with Applications*.

[37] Schweizer B, Sklar A (1983). *Probabilistic Metric Spaces*.

[38] Klir G, Yuan B (1995). *Fuzzy Sets and Fuzzy Logic: Theory and Applications*.

[39] Nguyen HT, Walker RA (1997). *A First Course in Fuzzy Logic*.

[40] Klement EP, Mesiar R (2005). *Logical, Algebraic, Analytic and Probabilistic Aspects of Triangular Norms*.

[41] Xu ZS, Sun ZD (2002). Priority method for a kind of multi-attribute decision-making problems. *Journal of Management Sciences in China*.

[42] Yue Z (2011). Deriving decision maker’s weights based on distance measure for interval-valued intuitionistic fuzzy group decision making. *Expert Systems with Applications*.

[43] Xia MM (2012). *Research on fuzzy decision information aggregation techniques and measures [Dissertation]*.

[44] Torra V (2010). Hesitant fuzzy sets. *International Journal of Intelligent Systems*.

